# Precision fMRI and cluster‐failure in the individual brain

**DOI:** 10.1002/hbm.26813

**Published:** 2024-08-26

**Authors:** Igor Fabian Tellez Ceja, Thomas Gladytz, Ludger Starke, Karsten Tabelow, Thoralf Niendorf, Henning Matthias Reimann

**Affiliations:** ^1^ Max‐Delbrück‐Center for Molecular Medicine in the Helmholtz Association (MDC), Berlin Ultrahigh Field Facility (B.U.F.F.) Berlin Germany; ^2^ Charité—Universitätsmedizin Berlin Berlin Germany; ^3^ Weierstrass Institute for Applied Analysis and Stochastics Berlin Germany; ^4^ Experimental and Clinical Research Center (ECRC), A Joint Cooperation between the Charité Medical Faculty and the Max‐Delbrück Center for Molecular Medicine in the Helmholtz Association Berlin Germany

**Keywords:** adaptive weights smoothing, BOLD, cluster failure, fMRI, single‐subject, spatial accuracy, spatial smoothing

## Abstract

Advances in neuroimaging acquisition protocols and denoising techniques, along with increasing magnetic field strengths, have dramatically improved the temporal *signal‐to‐noise ratio* (tSNR) in functional magnetic resonance imaging (fMRI). This permits spatial resolution with submillimeter voxel sizes and ultrahigh temporal resolution and opens a route toward performing precision fMRI in the brains of individuals. Yet ultrahigh spatial and temporal resolution comes at a cost: it reduces tSNR and, therefore, the sensitivity to the *blood oxygen level‐dependent* (BOLD) effect and other functional contrasts across the brain. Here we investigate the potential of various smoothing filters to improve BOLD sensitivity while preserving the spatial accuracy of activated clusters in single‐subject analysis. We introduce *adaptive‐weight smoothing with optimized metrics* (AWSOM), which addresses this challenge extremely well. AWSOM employs a local inference approach that is as sensitive as cluster‐corrected inference of data smoothed with large Gaussian kernels, but it preserves spatial details across multiple tSNR levels. This is essential for examining whole‐brain fMRI data because tSNR varies across the entire brain, depending on the distance of a brain region from the receiver coil, the type of setup, acquisition protocol, preprocessing, and resolution. We found that cluster correction in single subjects results in inflated family‐wise error and false positive rates. AWSOM effectively suppresses false positives while remaining sensitive even to small clusters of activated voxels. Furthermore, it preserves signal integrity, that is, the relative activation strength of significant voxels, making it a valuable asset for a wide range of fMRI applications. Here we demonstrate these features and make AWSOM freely available to the research community for download.

## Graphical abstract

Functional magnetic resonance imaging (fMRI) at ≥7 T achieves finer spatial resolution, eventually causing a decrement in temporal signal‐to‐noise ratio and thus blood oxygen level‐dependent (BOLD) sensitivity. Adaptive‐weight smoothing with optimized metrics (AWSOM) enhances BOLD effects detection with high spatial accuracy and preserves the integrity of BOLD signal magnitudes. AWSOM minimizes family‐wise error rates by effectively suppressing false positives in single‐subject fMRI analysis.

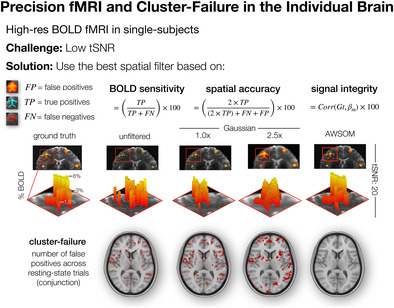

**Practitioner Points**

- Ultrahigh field functional magnetic resonance imaging (fMRI) (≥7 T) allows for submillimeter spatial resolution acquisitions. This can reduce temporal signal‐to‐noise ratio and sensitivity to blood oxygen level‐dependent (BOLD) effects.
- Adaptive‐weight smoothing with optimized metrics (AWSOM) enhances BOLD sensitivity while preserving the spatial accuracy of detected voxel clusters. AWSOM utilizes a local smoothing approach that balances sensitivity with preserving spatial detail and signal integrity of activated voxels.
- AWSOM effectively suppresses false positives by controlling family‐wise error rates in single‐subject fMRI, which is not the case for cluster correction. It is made freely available for the research community.

## 1 INTRODUCTION

Functional magnetic resonance imaging (fMRI) is a mainstay of neuroscience. The advent of ultrahigh field MRI (UHF, B_0_ ≥ 7 Tesla), improvements in pulse sequences (Huber et al., 2018; Huber et al., 2021) and denoising techniques (Beckers et al., 2023; de Cheveigné & Nelken, 2019; Vizioli et al., 2021), have significantly enhanced the sensitivity to the *blood oxygen level‐dependent* (BOLD) effect (Ogawa et al., 1992) and other neurovascular contrasts used in fMRI (Dowdle et al., 2023; Huber et al., 2021). This progress has ushered in a new era in fMRI, enabling examinations of individual subjects' brains at submillimeter spatial resolution (Harmer et al., 2012; Yacoub et al., 2001). This advancement is crucial for identifying fine‐grained neural circuits and elucidating their roles in complex cognitive functions and neurological disorders (De Martino et al., 2018; Uğurbil, 2018).

The superior resolution of UHF fMRI permits the capture and analysis of the intrinsic details of activated neurovascular units down to the cortical layer level (Shamir et al., 2022; Viessmann & Polimeni, 2021) and the depths of brain stem nuclei (Gizewski et al., 2014; Sclocco et al., 2018; Uğurbil, 2021). The ability to resolve the unique aspects of functional activity in the individual brain permits the profiling of functional signatures and an understanding of neural mechanisms and their variations (Besle et al., 2013; Dubois & Adolphs, 2016), often masked out in group‐level analyses (Feredoes & Postle, 2007). An individualized approach is necessary to enhance the precision of cognitive and neurological research and provides a springboard for personalized treatments and interventions in clinical settings (Chen et al., 2012; Jabakhanji et al., 2022).

At the same time, high spatial resolution fMRI is challenged by the *temporal signal‐to‐noise ratio* (tSNR) due to the reduction of the image voxel size in order to further nominal spatial granularity (Tabelow et al., 2009; Vizioli et al., 2021). This lowers the BOLD sensitivity (BS) and raises the detection threshold for profiling subtle neurovascular activity (Runge et al., 2007; Zhu et al., 2022). The tSNR is a fundamental metric in fMRI, reflecting the precision with which the BOLD signal can be discerned from the background noise over time (Liu, 2016; Murphy et al., 2007; Welvaert & Rosseel, 2013). The spatial aspect of noise involves high‐frequency random fluctuations and lower‐frequency drifts that obscure the BOLD signal, eventually leading to a reduced identification of brain activity patterns or even incorrect results (Caballero‐Gaudes & Reynolds, 2017; Greve et al., 2013). By enhancing tSNR, a more accurate representation of the BOLD signal can be achieved (Tabelow et al., 2009; Van Der Zwaag et al., 2012; Vizioli et al., 2021), which is essential for reliable detection and localization of brain activity, particularly when exploring fine‐grained neuroanatomical structures in the individual brain.

So there is a need for spatio‐temporal filtering in high‐fidelity fMRI. In pursuit of reliability, it is also essential to discriminate true neurovascular activation from false positive (FP) signals (Eklund et al., 2016). This distinction remains to be established with single‐subject fMRI, as FPs may be averaged out in group analysis but not in a single brain. Compounding the problem, higher resolutions have been shown to inflate the FP rate (FPR; Mueller et al., 2017). This work examines approaches to smoothing and statistical inference regarding their ability to enhance tSNR while preserving the fine‐grained spatial details that are crucial for accurate brain activity mapping in UHF fMRI in the individual subject. To meet this need, we conducted an in‐depth evaluation and validation of three types of spatial smoothing.

First, we examined the *Gaussian smoothing* for cluster‐corrected statistical inference, which is the most common approach in fMRI analysis (Eklund et al., 2016; Woo et al., 2014). This method is known to enhance BS (Woo et al., 2014). Gaussian smoothing employs the convolution of the image with a Gaussian kernel function with a fixed size (bandwidth) to reduce noise (Kruggel et al., 1999; Mikl et al., 2008). This approach has a significant drawback, however, due to its tendency to blur fine spatial details, including anatomical boundaries. This can lead to the inclusion of confounding effects from neighboring non‐active voxels. Gaussian blurring may be beneficial at the group level by increasing the overlap of “representative” population effects. Single‐subject fMRI respects the divergent architectures and functionalities across brains (Westlin et al., 2023), focusing on the fine‐grained idiosyncrasies of individual processing structures in pursuit of resolving cortical layer profiles or specific subdivisions of brainstem nuclei. To mitigate spatial blurring, we specifically chose small Gaussian kernel sizes of 1× and 1.5× times the spatial resolution, aiming to reduce noise while preserving as much spatial detail as possible (Friston et al., 1995; Mikl et al., 2008). This approach was contrasted with the traditional use of a Gaussian kernel at 2.5× the spatial resolution (Liu et al., 2001; Poldrack et al., 2011). While this is effective at noise reduction, it is known to heavily blur fine details, potentially attenuating critical neurofunctional information.

Second, the *spatial adaptive nonlocal means* (SANLM) filter (Manjón et al., 2010) has been introduced into fMRI to locally smooth the data while preserving fine‐grained details (Bernier et al., 2014). Here, we test its suitability for cluster correction as an alternative to Gaussian smoothing. The SANLM filter reduces noise by examining each voxel in the image, finding voxels with similar intensity values throughout the entire brain volume, and then averaging these to smooth out the noise (Bernier et al., 2014; Tellez Ceja et al., 2022). SANLM was originally intended to denoise anatomical MR images; by leveraging information redundancy in spatially distributed patterns, the SANLM filter aims to maintain the integrity of edge details while reducing noise, with the intensity levels governing the trade‐off between detail preservation and noise reduction (Manjón et al., 2010). In fMRI, activation patterns exhibit a higher level of detail with SANLM compared to Gaussian smoothing (Bernier et al., 2014; Tellez Ceja et al., 2022). Yet, it is not clear whether these patterns correspond to underlying BOLD effects or whether they are biased by local anatomical contrasts.

The *adaptive weights smoothing* (AWS) filter emerged as an evolution of spatial adaptive fMRI filters (Tabelow et al., 2006). As SANLM, the AWS filter focuses on finding a balance between noise suppression and spatial resolution. AWS stands apart from Gaussian and SANLM filters by smoothing the statistical parametric maps (SPMs) instead of the original volumes of the time series. This contrasts with cluster correction, which relies on a uniform Gaussian smoothness of the data to determine the significance of voxel clusters. Instead, AWS defines locally adaptive weighting schemes that are based on local differences in the data: it incorporates information on spatial correlation in the data by considering the residuals obtained by the general linear model (GLM) in unfiltered data (Tabelow et al., 2011). In addition to exploring the AWS filter in default settings, we examine optimal parameter settings for AWS to customize to the specific demands of decoding fine‐grained functional signatures in the individual brain at 7 T, which we termed AWS *with optimized metrics* (AWSOM).

This study systematically evaluates and compares the performance of Gaussian, SANLM, and AWS filters, compared to unfiltered data, as a means of finding an optimal balance between noise reduction and the preservation of high‐resolution detail in UHF fMRI. We developed an analytical framework, that employs a synthetic fMRI dataset as a ground truth. This framework allows us to evaluate how effectively different filter types preserve BOLD‐related information by using three metrics: BS, reflecting BOLD effect detectability across tSNR levels; spatial accuracy (SA) of activated voxel clusters; and signal integrity (SI) of the hemodynamic response function (HRF) based on magnitude distribution within these clusters. We applied our insights in a proof‐of‐principle pilot study where we acquired and analyzed task‐based 7 T fMRI data for a finger‐tapping paradigm. Finally, we performed a null‐hypothesis test using resting‐state 7 T fMRI data, for which we modeled different block and event‐related designs, as proposed by Eklund et al. (2016), to determine the *family‐wise error rate* (FWER), and FPR produced by each filter for data obtained from in the brains of individual subjects.

Our work contributes to the fields of neuroimaging and neuroscience by providing an in‐depth analysis and demonstration of the precision and efficacy of fMRI smoothing filters and their respective FPR for 7 T fMRI in the individual brain. The improved AWS filter outperforms previously available filters by enhancing tSNR across the brain without sacrificing the high‐resolution details of 7 T fMRI. *En route* to unveiling fine‐grained functional neurosignatures at the single subject level, we share the advanced AWSOM filter with the community in an open access git repository.

## 2 METHODS

### 2.1 Synthetic datasets

The creation of synthetic fMRI datasets involved several steps. First, a single volume from a task‐based fMRI dataset acquired at 7.0 T (single shot EPI, TR/TE = 1200/33.2 ms, spatial resolution = 1.5 mm isotropic) was selected and duplicated 599 times as anatomical reference for the synthetic time series. Next, four spatial masks with different shapes and sizes were created to delineate the regions of activation (ROA), as ground truth. Then, eight activation onsets, each separated by an interstimulus time of 90 s, were convolved with a double gamma function (Friston et al., 1998; Glover, 1999) to simulate the HRF. The HRF was set with a time to peak of 4.8 s, an initial dip at 1.2 s, poststimulus undershoots at 8.4 s, and return to baseline after 20 s since the onset. Two types of noise distributions were simulated: Gaussian and Rician. Gaussian noise was generated in Python using a function to produce pseudorandomized values matching the data's dimensions, each with a variance set to 1. Rician noise was computed as the square root of the sum of two squared and independent Gaussian noises.

To explore the filter's efficiency at different noise levels, the two types of noise were amplified to five magnitudes, 1, 2, 4, 8, and 16%, the percentages are relative to the baseline. Finally, the noises were added to the synthetic BOLD signal time series.

Two types of data sets were designed with ROAs comprising either homogeneous or heterogeneous BOLD magnitudes. For homogeneous ROAs, the same magnitude in all voxels was set within the ROAs. The BOLD magnitudes used were 0.5, 1, 2, 3, 4, 5, and 6%. For heterogeneous ROAs, three BOLD magnitudes were mixed within the ROA, 1.5, 3, and 6%. Ten time series were created for each noise level, BOLD magnitude, and type of noise distribution. The results shown are based on the average of the time series, with standard deviation represented by error bars.

The time series were simulated across all five noise levels. However, due to the negligible detection of activations at higher noise levels (8 and 16%), the comparison of filter performance in the results section is limited to data up to the 4% noise level.

### 2.2 Data processing and analysis

Preprocessing was performed using *NeuroImaging PREProcessing toolS* (fMRIPrep) (Esteban et al., 2019), which included structural MRI procedures: brain extraction, tissue segmentation, and spatial normalization. For BOLD sequences, preprocessing involved image estimation, head motion correction, slice time adjustment, susceptibility distortion correction, and registration to anatomical and standard spaces.

Gaussian smoothing was carried out at 1.5, 2.5, and 3.5 full width at half maximum millimeter, which correspond to kernel sizes of 1×, 1.5×, and 2.5× times the voxel resolution. The *Analysis of Functional NeuroImages* (Cox, 1996) tool was employed for Gaussian filtering. FSL FEAT was used for first‐level analysis by GLM regression. Statistical inference was conducted based on cluster correction with a threshold of 3.1 and a *p*‐value of <.05.

SANLM filtering of fMRI data was performed via the *Computational Anatomy Toolbox* (Gaser et al., 2022) in SPM (Penny et al., 2007) 12 at three intensities: light, medium, and strong. The filter strength in the context of the nonlocal means denoising algorithm, as discussed by Manjón et al. (2010) is regulated by the parameter ℎ^2^. This parameter determines the degree of smoothing applied during the denoising process. Essentially, ℎ^2^ controls how much influence neighboring voxels have on the denoised value of a given voxel of interest. A higher value of ℎ^2^ results in stronger smoothing because it increases the weight of the differences between intensity values in the averaging process, leading to more aggressive noise reduction. The optimal value for ℎ^2^ has been experimentally found to be *σ* ^2^, where *σ* represents the standard deviation of the noise (Buades et al., 2008; Lee, 1980). This setting is based on the assumption that the noise is stationary (uniform across the image). In this scenario, a global variance (*σ* ^2^) effectively guides the smoothing process, balancing noise reduction against the preservation of image details. The SANLM algorithm estimates the ℎ^2^ value to be used based on the selected filtering strength. The medium strength performs the nonlocal adaptive filtering less intensively than the standard form. Medium uses a single iteration and evaluates the possibility of applying low resolution filtering, which down samples the volume to the double resolution size, filters and then resamples to the original resolution. This option is only applied on submillimeter data. The light strength filters with the half intensity of the medium strength, but without applying the sub‐resolution filtering. The Strong option uses the full adaptive nonlocal filter strength, forces sub‐resolution filtering and applies an additional iteration (Gaser et al., 2022). Statistical inference was conducted based on cluster correction with a threshold of 3.1 and a *p*‐value of <.05.

The AWS filter runs in *R* environment, as part of the *fmri* and *aws* libraries (Polzehl et al., 2020; Tabelow et al., 2011). AWS employs the contrast beta weights, also called contrast parameter estimates (COPEs), variance contrast beta weights, and the residuals as inputs. Those files were computed by the FSL GLM analysis (Jenkinson et al., 2012; Smith et al., 2004). AWS filter operates through an iterative process that utilizes a series of incrementally increasing bandwidths. These bandwidths establish adaptive, or data‐dependent, weighting schemes for conducting local averaging within specified vicinities. The calculation of weights within these vicinities incorporates two components: a traditional, non‐adaptive kernel filter part, and an additional factor that assesses the discrepancy in values between two voxels. This assessment is based on the extent of variance reduction achieved in preceding iterations. Consequently, this iterative process leads to a progressive decrease in the variance of the estimated denoised values, enhancing the signal's clarity. To prevent the inclusion of outlier values in the local averages, the AWS method automatically reduces the weights, thereby excluding such outliers from the averaging process. The steering parameter in this procedure is *λ*, which governs the adaptation process. A high *λ* value, approaching infinity, reverts the process to that of classical, non‐adaptive kernel smoothing like the Gaussian filter, while a *λ* value close to zero maintains the data in its original, unaltered state. The selection of the optimal *λ* value is not contingent upon the specific dataset but is determined prior to analysis, based on the anticipated noise distribution. It is important to note, however, that inaccuracies in the assumed noise distribution or the presence of spatial and temporal correlations may necessitate fine‐tuning of the *λ* parameter.

**FIGURE 1:**
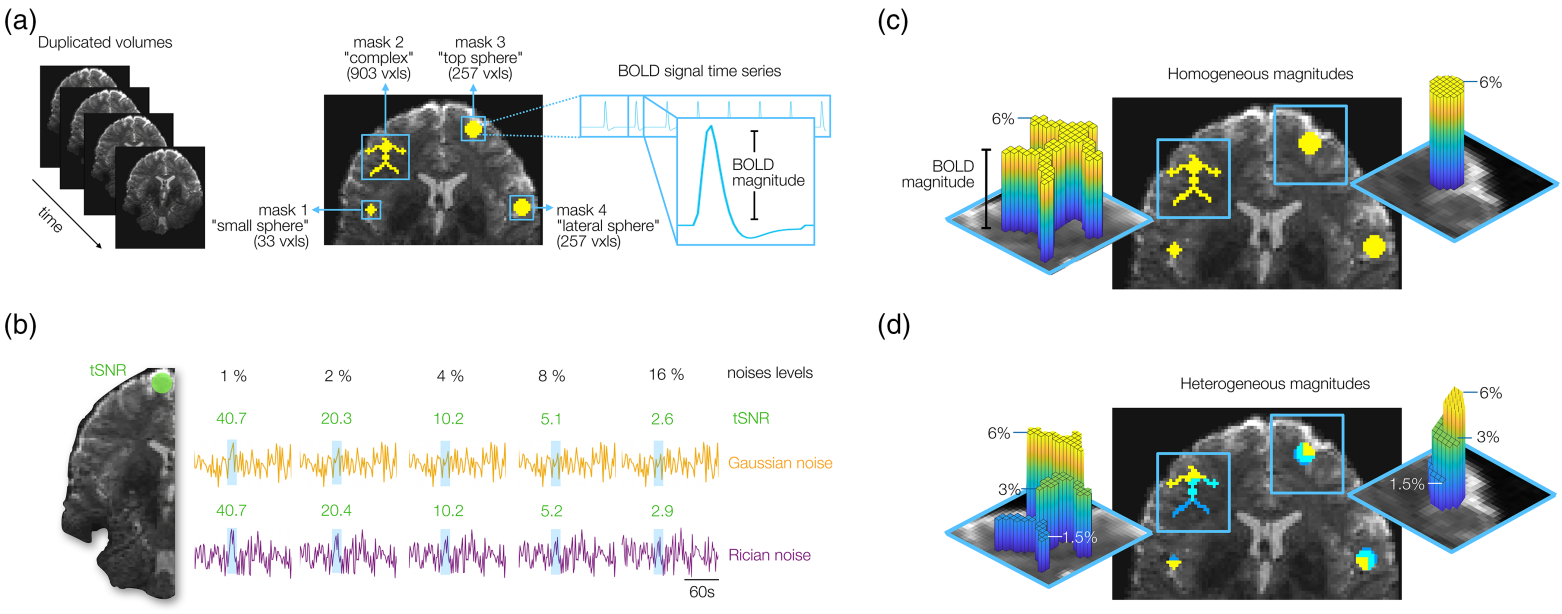
Synthetic fMRI time series creation. (a) Four different masks were created to define the regions of activation (ROA) as ground truth. The voxels within the activation regions were convolved with a double gamma function to model the hemodynamic response function (HRF). (b) Gaussian and Rician noise distributions were simulated and amplified to five magnitudes (1–16%). To contextualize the simulated noise levels, the temporal signal‐to‐noise ratio (tSNR) was measured in the frontal lobe of the simulated time series, indicated in green. The tSNR values observed for varying noise levels were consistent for both noise distributions: 40 at 1% noise, 20 at 2%, 10 at 4%, 5 at 8%, and 2 at 16%. The noises were added to the synthetic BOLD signal time series. Two types of datasets were created: One with homogeneous BOLD magnitudes for each level, and another heterogeneous, combining the three magnitudes. (c) Homogeneous magnitude: The simulated BOLD magnitude was the same across all the voxels in the ROA. Seven magnitudes (0.5, 1, 2, 3, 4, 5, and 6%) were used for each simulation. (d) Heterogeneous magnitudes: Three magnitudes were combined on each mask (1.5, 3, and 6%).

The *λ* value for AWSOM is refined by reducing it by 20% from the default AWS setting (from *λ* = 1 to *λ* = 0.8). This adjustment enhances the method's sensitivity to detect smaller activated clusters while concurrently minimizing FPs. A *λ* of 0.8 is estimated to be the threshold for detecting small clusters at an intermediate noise level (2%), such as mask 1 with a size of 33 voxels (Figure 1a). This resulted in a reduction in SA and SI of less than a 2%. Reducing *λ* by more than 20% would significantly increase the number of FPs, thereby adversely affecting accuracy and SI—key metrics that we strive to maintain. Furthermore, AWSOM is distinct from AWS by generating statistical parametric output maps that show the magnitude of the BOLD signal at the statistically significant voxels. These are generated by applying a mask of Z maps to the COPE file.

AWS and AWSOM filters are based on the propagation‐separation approach proposed by Polzehl et al. (Polzehl & Spokoiny, 2005) and later suggested for fMRI analysis (Polzehl et al., 2010; Tabelow et al., 2006). Further information on the filter operation is described by Tabelow et al. (Polzehl & Tabelow, 2019; Tabelow et al., 2009). AWS and AWSOM have been extracted from R and binaries have been compiled for use under Bash and Python. We publish the pipelines, scripts, and packages for download in our GitHub repository.

### 2.3 Evaluation metrics

All the active voxels that coincide with the location of the ground truth are considered true positives (TPs). Similarly, all voxels that are not part of the ground truth are true negatives. All the non‐active voxels that are within the ROA are false negatives (FNs), and all the active voxels outside the ROA are FPs. The SA was calculated with the *Sørensen‐Dice* coefficient formula, defined as two times TP divided by the sum of two times TP plus FN plus FP and multiplied by 100, as seen in (1). BS: TP divided by the sum of TP and FN and multiplied by 100, as seen in (2). SI: is calculated as the correlation between the ROA, used as ground truth (*Gt*) in space and magnitude, and the COPEs $\left(\beta_{m}\right)$, calculated from the GLM, and masked with the thresholded activity map (*z* map), and multiplied by 100, as seen in (3). If there were no identified activations to compare, the correlation was designated as 0.

(1) $$\mathrm{SA}=\left(\frac{2\times \mathrm{TP}}{\left(2\times \mathrm{TP}\right)+\mathrm{FN}+\mathrm{FP}}\right)\times 100$$

(2) $$\mathrm{SA}=\left(\frac{\mathrm{TP}}{\mathrm{TP}+\mathrm{FN}}\right)\times 100$$

(3) $$\mathrm{SI}=\text{Corr}\left(\mathrm{Gt},\beta_{m}\right)\times 100$$

The results of the three metrics are presented in three‐dimensional graphs. This allows for a comprehensive comparison of the filters, including the average values across trials and their respective standard deviations. The averaged values are additionally displayed in numbers as heat maps in the supplementary Figures [Link], [Link].

### 2.4 Task‐based fMRI data

Three right‐handed, healthy participants were included after receiving approval by the local ethical committee. Informed, written consent was obtained from each volunteer before the study. A finger‐tapping paradigm was employed, which consisted in tapping each finger (index, middle, ring, and small) against the thumb of the right hand, with an approximate frequency of 3 times/second.

The tapping activity was requested for 30 s for each finger, and 15 s of rest between fingers. Tapping the 4 four fingers consisted of one cycle. The run consisted of 5 cycles, with a total time of 15 min and 15 s, including 15 s of rest before the first tapping.

The fMRI time series (GE‐EPI, TR/TE/FA = 2000 ms/28 ms/66°, FOV/matrix = 240 × 244 mm/160 × 160, 80 slices, 1.5 mm isotropic, 458 volumes) were acquired on a MAGNETOM 7 T scanner (Siemens Healthcare, Erlangen, Germany) with a single‐channel‐ transmit/32‐channel receive head coil (Nova Medical, Wilmington, MA, USA). The datasets were pre‐filtered with NORDIC (Vizioli et al., 2021), preprocessed with fMRIPrep (Esteban et al., 2019), filtered and processed using the same software tools that were used for the synthetic datasets.

### 2.5 Resting‐state fMRI data

Seven healthy participants underwent two resting‐state fMRI scans within a single session, while two additional healthy participants were subjected to only one resting‐state fMRI scan each. All subjects were included after receiving approval by the local ethical committee. Informed, written consent was obtained from each volunteer before the study. This resulted in a total of 16 time series. Participants received no specific instructions during the scanning process. Each scanning session lasted for 10 min.

The resting‐state fMRI time series (GE‐EPI, TR/TE/FA = 2000 ms/28 ms/66°, FOV/matrix = 240 × 264 mm/160 × 160, 80 slices, 1.5 mm isotropic, 300 volumes) were acquired on a MAGNETOM 7 T scanner (Siemens Healthcare, Erlangen, Germany) with a single‐channel‐ transmit/32‐channel receive head coil (Nova Medical, Wilmington, MA, USA). The datasets were preprocessed with fMRIPrep (Esteban et al., 2019) and denoised using FSL's Melodic (Jenkinson et al., 2012; Smith et al., 2004). First‐level analyses with FSL FEAT (Jenkinson et al., 2012; Smith et al., 2004), and registration with ANTs (Avants et al., 2011).

We used the acquired resting‐state data to investigate FPR in task‐based fMRI for the smoothing filters that were applied. Following the protocol of Eklund et al. (2016), we analyzed the resting‐state data as if it were task fMRI, where no activations should ideally be present, modeling four different paradigms. Any detected activations were considered FPs, indicating the unreliability of statistical inference under the tested conditions. Eklund et al. (2016) used this approach to demonstrate inflated FPRs produced by different software packages in fMRI on a group level; we here focus on the FPRs obtained for different smoothing filters in single‐subject fMRI at high resolution. Four paradigms were designed, two block activity paradigms (B1 and B2) and two event‐activity paradigms (E1 and E2), as used by Eklund et al. (2016). The B1 paradigm consisted of 10‐s on and 10‐s off cycles. B2 in cycles of 30‐s on and 30‐s off. The E1 event activity paradigm consisted of 2 s of activity and 6 s of rest. E2 between 1 and 4 s of activity and between 3 and 6 s of rest, in a pseudorandom manner. In our study, to increase the number of analyses and thus statistical power, we designed variations of each of the paradigms. We shifted the activity times by 5 s for B1 and B2, creating four paradigms for B1 and 13 for B2. Following the same logic, E1 was shifted by 4 s, creating an additional paradigm. With E2 we took advantage of the use of the pseudorandom function of its activity and rest times, creating 16 paradigms. With the 16 time‐series and the paradigm variations, 64 analyses were performed with B1, 208 with B2, 32 with E1 and 256 with E2. This gives a total of 560 different analyses for each filter.

The FWER was calculated by dividing the number of analyses with at least one active voxel by the total number of analyses of each paradigm type. The FPR was calculated as the number of active voxels divided by the total number of voxels in the brain, averaged over all analyses of each paradigm type. In addition, we calculated the mean number of active clusters and the mean number of active voxels for each paradigm type. FP distribution maps were produced by calculating the sum of all thresholded binarized z‐maps for each filter and for each paradigm type, after being registered to standard MNI space (MNI152NLin6Asym).

## 3 RESULTS

### 3.1 Overview of the synthetic data sets

The synthetic fMRI dataset, with an isotropic voxel size of 1.5 mm, exhibited four ROA with diverse shapes and sizes that served as our ground truth, in which HRFs were modeled as BOLD signal changes over time with magnitudes ranging from 0.5% to 6% (Figure 1a). To simulate different tSNRs acquired for different MRI setups Gaussian and Rician noises were added to the synthetic BOLD signal time series at a broad range of magnitudes (Figure 1b). Higher noise levels were presumed to increasingly obscure the HRFs, challenging their detection by the GLM regression model. Most evaluations were performed based on datasets with homogeneous ROAs comprising similar BOLD magnitudes across all voxels (Figure 1c). To model a more realistic scenario, a dataset with heterogeneous ROAs contained a mix of three magnitudes—1.5, 3, and 6% (Figure 1d). We used this dataset to evaluate the filters' abilities to preserve the integrity of relative BOLD magnitudes at a later stage of our study.

### 3.2 Comparative analysis of filtering effects on BOLD activity

**FIGURE 2:**
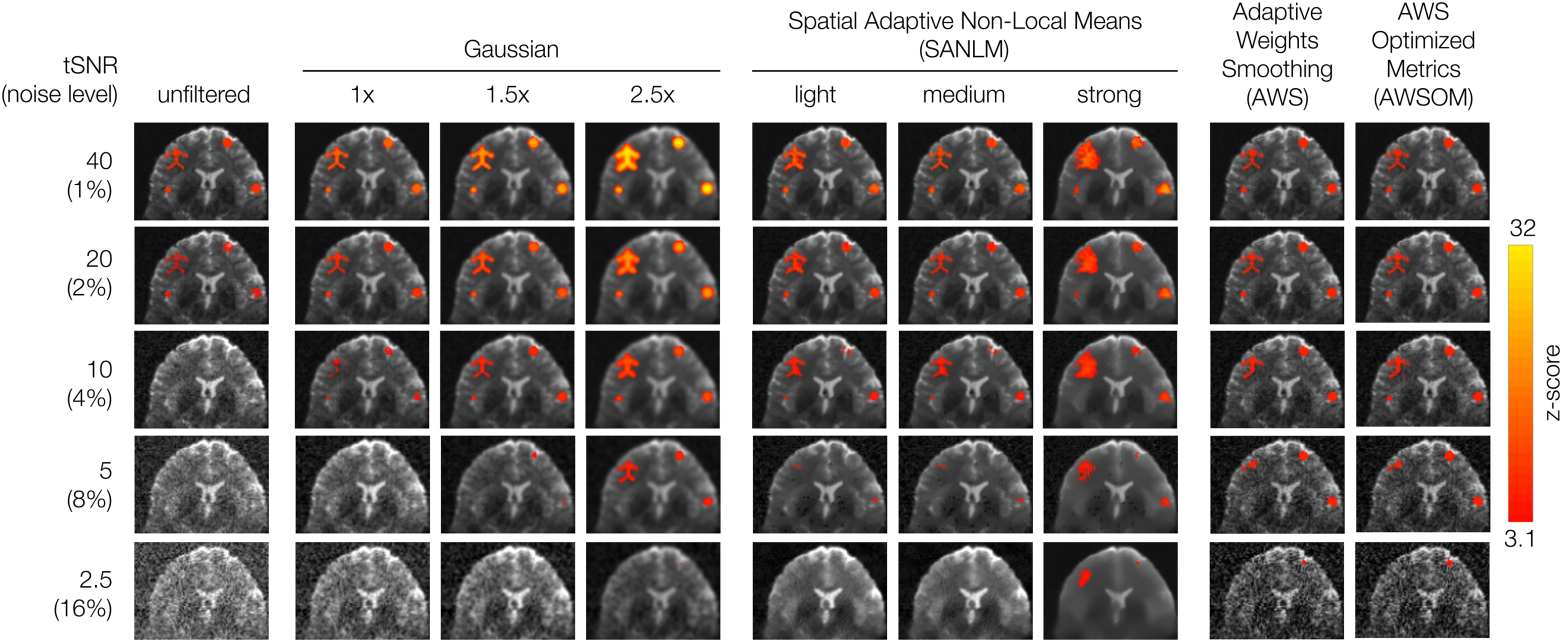
Averaged BOLD activity cluster comparison across 10 synthetic datasets with homogeneous magnitude. Gaussian noise at 1, 2, 4, 8, and 16% noise levels, 6% BOLD. Unfiltered data compared to Gaussian and SANLM and AWS filters. Gaussian smoothing amplifies the BOLD effect but blurs the spatial details of BOLD clusters. SANLM filtered data (especially strong SANLM) show amplification of BOLD effects, deforming the shapes of the original BOLD clusters. AWS and AWSOM showed similar results. Both preserved the geometry of the BOLD cluster at 1 and 2% noise levels. The BOLD shapes are distorted for higher noise levels for all filter methods. Gaussian and SANLM filters z‐scores decrease extensively for greater noise levels. Both AWS filters keep the same range of z‐scores along all the noise levels, depicting all the BOLD clusters with their highest value (≈6). AWS and AWSOM z‐scores are not comparable to those of other filters due to their specificities in the calculation. Nevertheless, they surpass the 3.1 threshold, after cluster correction.

We first investigated the distinct effects of the applied filtering methods on BOLD activity clusters within the synthetic data set, with noise levels ranging from 1 to 16%. The results were so similar for all noise types that we continue to show results only for Gaussian noise in the manuscript (Figure 2). The results for the Rician noise are to be found in the supplementary section (Supplementary Figures [Link], [Link]).

Gaussian smoothing filters enhanced the BOLD effect but at the cost of spatial detail within BOLD clusters. This effect became pronounced with increasing kernel size, with larger kernel sizes of 1.5× and 2.5× voxel sizes depicting the shape of the ROAs more accurately at higher noise levels. Data filtered with SANLM showed an amplified BOLD effect. However, this amplification resulted in an arbitrary deformation of the original shapes of the BOLD clusters with increasing filter strength. The AWS and AWSOM filters, on the other hand, effectively preserved the geometry of the ROAs at lower noise levels (1 and 2%). At higher noise levels, all filtering methods, including AWS, led to distortion in the shapes of ROAs and failed to identify active voxels.

Gaussian and SANLM filters exhibited a decrease in z‐scores as the noise levels increased. In contrast, AWS and AWSOM filters maintained a consistent range of z‐scores across all noise levels, depicting all BOLD clusters at their highest value (6.36). It is important to note that the z‐scores obtained from AWS filtering are not directly comparable to those from other filters due to differences in calculation methodologies. Nevertheless, these z‐scores exceeded the threshold of 3.1, corresponding to a *p*‐value of .001 after cluster correction, indicating their statistical significance.

### 3.3 Metrics for evaluating simulated fMRI data

**FIGURE 3:**
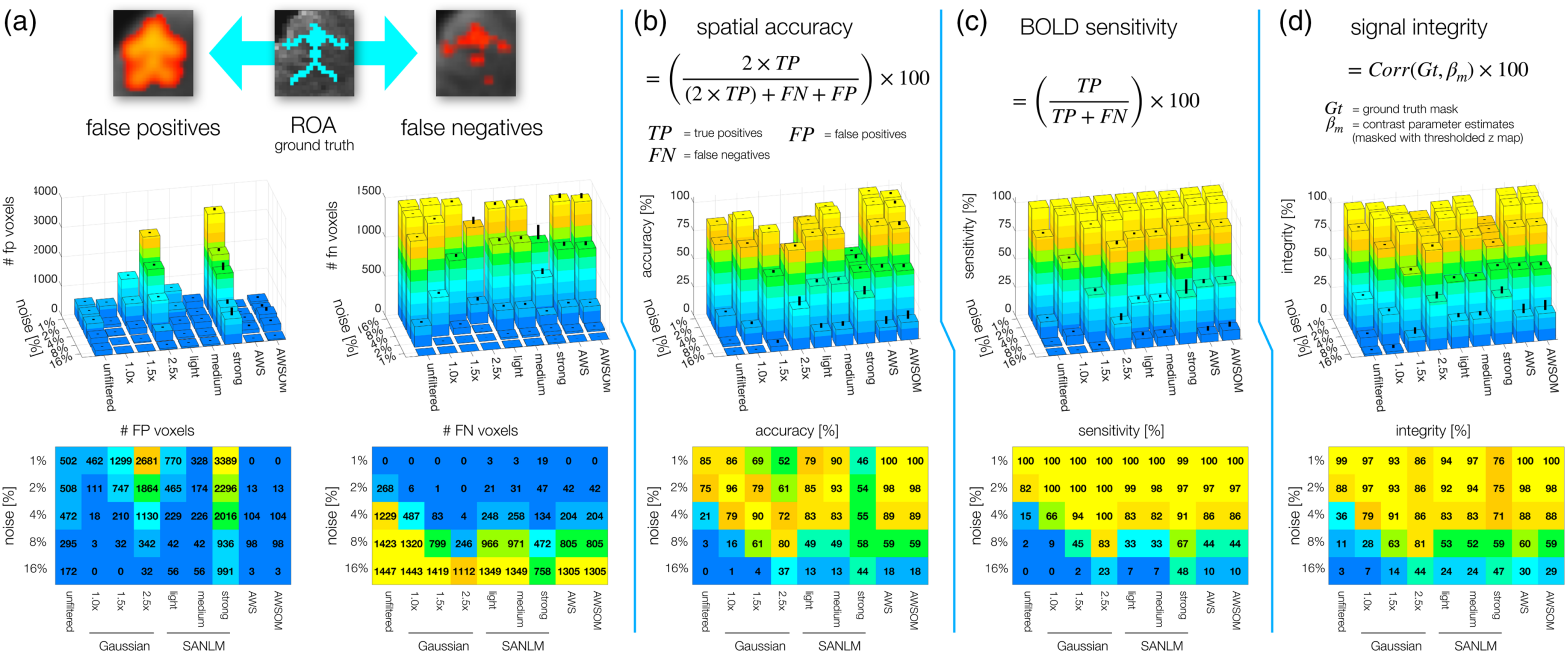
Simulated time series evaluation metrics. BOLD homogeneous magnitude of 6%, five levels of noise (1, 2, 4, 8, and 16%) with Gaussian noise distribution, assessing the whole brain. To calculate the spatial accuracy and BOLD sensitivity, the number of false negatives (FN), false positives (FP), and true positives (TP) were quantified. (a) First row: Graphical description of FP and FN compared to the ROA mask 2. All voxels that presented activations that were not active in the synthetic datasets are considered FP. The missing active voxels within the ROA are FN. Second row: Number of FP and FN voxels counted, using each filter (horizontal axis) for each noise level (vertical axis) displayed in 3D. The black bars above represent the standard deviation. Third row: Heat maps presenting the same information as in the second row, but in 2D. (b) Spatial accuracy (SA) was calculated using the Sørensen‐Dice coefficient formula (first row) and multiplied by 100 to obtain values in percentages (second row). The SA for the five noise levels depicts low values to the SANLM strong filter and Gaussian 1.5× and 2.5× kernel sizes due to the inflated FP. AWS and AWSOM obtained the same results; they achieved the highest spatial accuracy. (c) First row: BOLD sensitivity (BS) formula in percentage. Second row: BS values. The BS was approximately similar between all the filters. (d) Signal Integrity (SI) is a metric defined to evaluate the effect of the filtering methods on the representation of relative activity strength within an activated region. The SI is calculated as the correlation between the ROA, used as ground truth in space and magnitude, and the contrast parameter estimates (COPEs), calculated from the general linear model (GLM) and masked with the cluster corrected activity z map. At 1% noise level, the distortion effects that the filters have on the BOLD signal become visible, indicating that denoising affects the signal. However, this is not the case for the AWS and AWSOME filters, which exhibit minimal distortion, thereby demonstrating their superiority.

To assess the filters' performances in preserving the BOLD signals for different noise levels, we evaluated them based on three metrics: the *sensitivity* to detect the BOLD signal change of activated voxels, the *SA* with which activated voxel clusters were preserved, and finally, the *integrity* of relative BOLD magnitudes within an activated voxel cluster. We initially focused on ROAs with a homogeneous BOLD magnitude of 6% across five noise levels ranging from 1 to 16%. To measure SA and BS, we quantified the number of FNs, FPs, and TPs across the entire brain. FPs are defined as voxels that have not been generated as showing BOLD signals over time in the synthetic datasets but have been detected as being activated, while FNs are active voxels within the ROAs that have not been detected as activated (Figure 3a).

BS, expressed in percentages, was calculated by the formula for statistical sensitivity (Figure 3b). At a noise level of 1%, corresponding to a tSNR of 40 (Figure 1b), all activated voxels have been detected across all filters, and even for unfiltered data we calculated a BS of 100%. The filters begin to effectively amplify lost BOLD signals at a noise level of 2% (tSNR of 20), where the BS of unfiltered data dropped to 82%, while BS was maintained at 97–100% across all filters. Although all filters' BS remained consistently high across the first three noise levels, the BS for 1× Gaussian filter dropped considerably at 4% noise (SNR of 10). The 2.5× Gaussian was the only filter to obtain a high value at 8% noise.

SA was determined using the *Sørensen‐Dice* coefficient formula, with results also converted to percentage values (Figure 3c). The resulting plots reflect the effects displayed in the example slice above (Figure 2). In contrast to the BS in unfiltered data of 100% at 1% noise, the SA in unfiltered data was only 85%, indicating the detection of FP across the brain when no filters were applied. The strong SANLM filter and Gaussian filters with 1.5× and 2.5× kernel sizes obtained the lowest SA values (70%<) at a noise level of 1%, attributed to an increase in FPs around the ROAs—emphasizing BS comes at the cost of SA for these filter types. Gaussian 1×, SANLM light and medium filters exhibited the highest SA at a noise level of 2%, Gaussian 2.5× at 8%, where most other filters, except AWS and AWSOM, failed to detect any activity. AWS and AWSOM showed the highest SA across all filters; they were the only ones that achieved an SA of 100% at a noise level of 1%, an SA of 98% at 2% noise, and still ranked among the highest SA with about 90% at a noise level of 4% together with Gaussian 1.5×. AWS was the only filter that produced a proportional decrease in SA as the noise level increased. This pattern would generally be expected and was also observed with unfiltered data, whose SA dropped to 75% at 2% noise and to 21% at 4% noise.

We also introduced SI as a metric to assess the impact of the filters on the representation of relative activity strength within an activated region. SI is calculated as the correlation between the ROA (used as ground truth in space and magnitude) and the COPEs derived from the GLM, for the voxels that have been found significant based on z statistics (z map), thresholded at 3.1 and cluster corrected (Figure 3d). This allowed us to assess BOLD magnitudes of statistically significant voxels. The distortion effects of the filters on the BOLD signal magnitudes were already evident at a noise level of 1%, indicating that all filters impact SI, with AWS and AWSOM filters appearing to be exceptions, preserving SI to 100%. Both AWS and AWSOM performed the best across all filters at noise levels of 1–2% and the second best at a noise level of 4%, preserving SI to 88%, slightly below Gaussian 1.5× with 91.

### 3.4 Detailed evaluation of individual ROA masks and the whole brain across BOLD magnitudes

In the previous sections, we found that the filters that were applied were feasible to principally preserve and amplify large BOLD effects of 6% magnitude across the first three noise levels, with a tSNR ranging from 40 to 10, the lowest. Below a tSNR of 10 (4% noise), all filters, except Gaussian 2.5×, heavily distorted the shape of complex ROAs or did not detect any signal at all. Therefore, we performed the following evaluations at the first three noise levels (1–4%≙tSNR 40–10), but for decreasingly lower BOLD magnitudes. We evaluated BS, SA, and SI individually for each ROA mask—comprising homogeneous BOLD magnitudes, as well as for the whole brain. The results described below correspond to the simulated noise with Gaussian distribution. Results for Rician noise are found in the supplementary section. Again, the results were consistent across the two types of noise.

**FIGURE 4:**
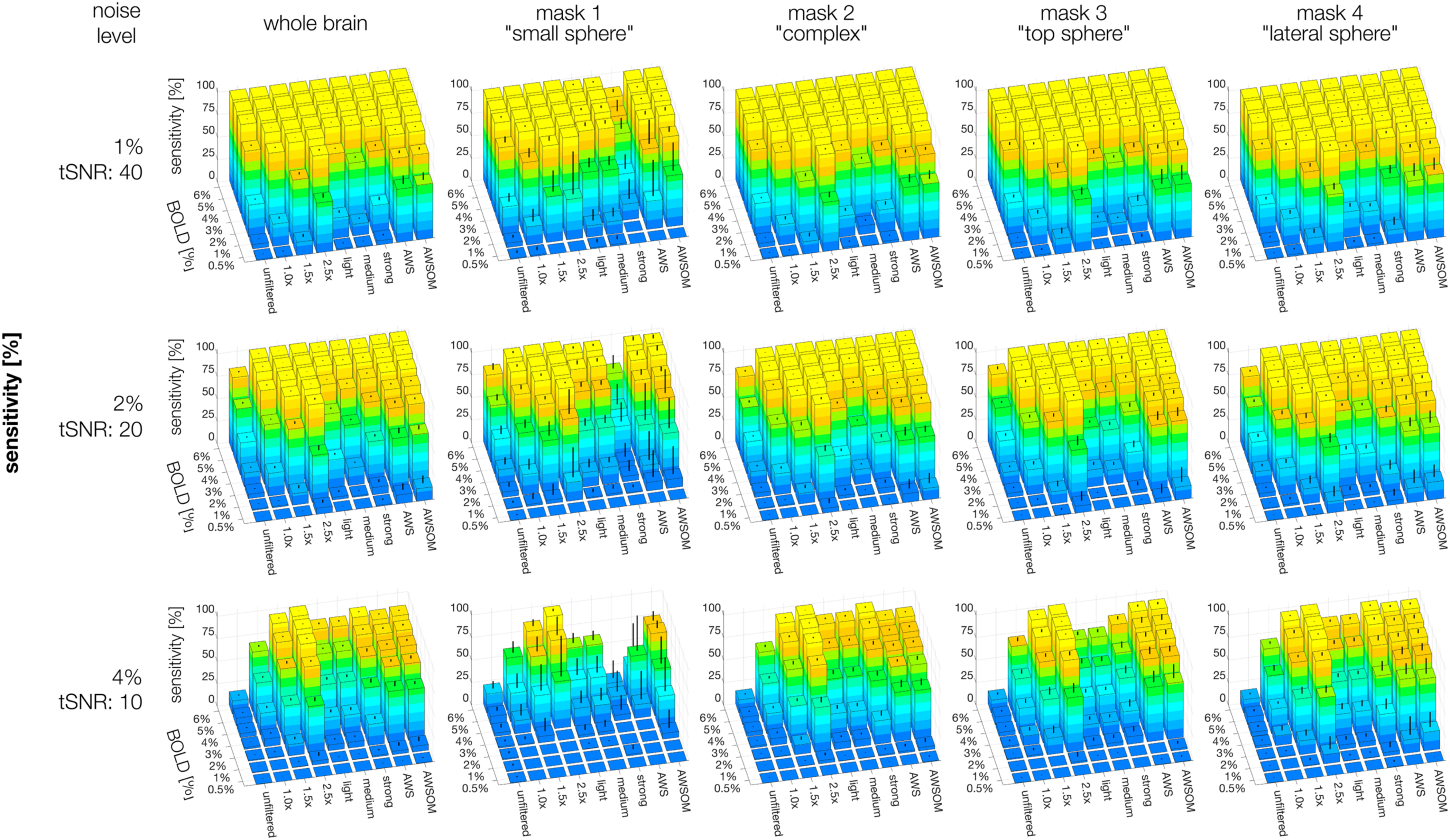
Sensitivity evaluated on simulated fMRI data sets using seven homogeneous BOLD magnitudes (0.5–6%) and three noise levels (1, 2, 4%) of Gaussian noise distribution. The error bars correspond to the standard deviation. The performance of all filters is comparably high at 1 and 2% noise levels. The 2.5× kernel size is shown as the best except for mask 1 at the 4% noise level, similarly as the accuracy results, the signal is removed along with the noise.

When noise levels were down to 1% (tSNR 40) and BOLD magnitudes around 3% and higher, BS was nearly perfect (≈100%) for all filters across all masks and the whole brain (Figure 4, supplementary Figure 1). Unfiltered data, however, suffered impaired BS already at BOLD magnitudes of 4%, while BS was down to one third for BOLD magnitudes of 3%, demonstrating that smoothing fMRI data can have tremendous effects on BS even at comparably high tSNR.

At 2% noise (tSNR 20), the filters that best amplified BS across the whole brain were Gaussian 2.5×, AWS and AWSOM (BS >80%). The lower the BOLD magnitude and the higher the noise level, the better these filters performed compared to others. This was true for the whole brain and all individual masks. However, for strong noise levels of 4% (tSNR 10), AWS performed decreasingly well for detecting the small spheric ROA of mask 1 at lower BOLD magnitudes, while Gaussian 2.5× suddenly failed to detect it for BOLD magnitudes of ≤3%. AWSOM, which we optimized to detect small clusters of subtle BOLD effects at low tSNR, preserved mask 1 with decent BS to BOLD magnitudes of ≥3%.

**FIGURE 5:**
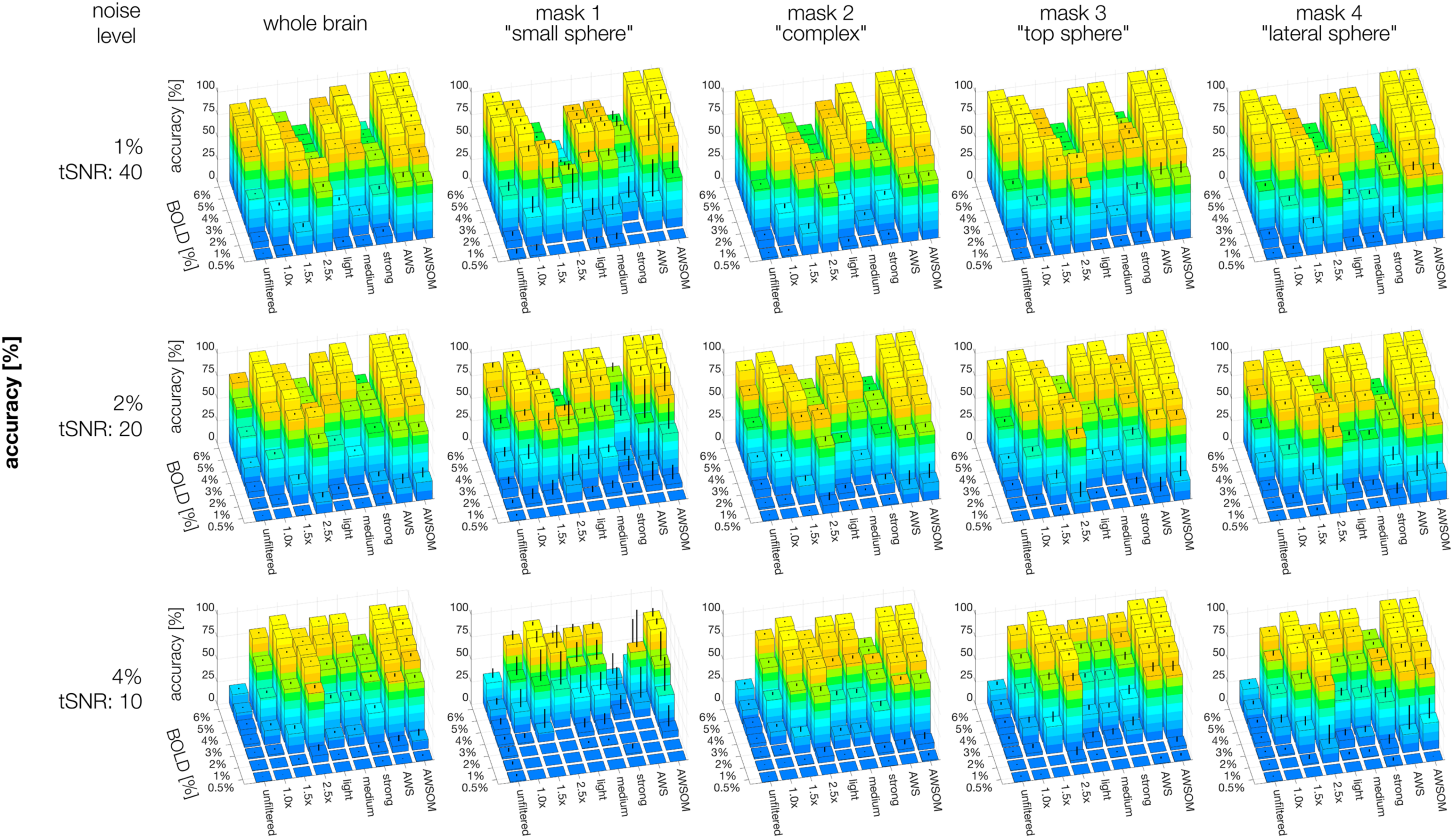
Accuracy evaluated on simulated fMRI data sets using seven homogeneous BOLD magnitudes (0.5–6%) and three noise levels (1, 2, 4%) of Gaussian noise distribution. The average of 10 simulations was calculated for each case. The error bars correspond to the standard deviation. The strong SANLM filter yielded the poorest results in all comparisons, followed by the Gaussian 2.5×. AWS and AWSOME performed the best for both whole‐brain analyses and for complex and large sphere clusters. The results for AWS and AWSOME are again similar, yet a notable difference emerges when comparing the outcomes for the small sphere cluster (mask 1). AWSOME demonstrates higher accuracy than both AWS and all other filters.

Mask 1 was the most challenging for the filters to detect, as it had the smallest cluster size, with 33 voxels. At 1% noise, decent SA results (>70%) were obtained from a BOLD magnitude of 2% for Gaussian 1× and 1.5×, light and medium SANLM, and AWS filters (Figure 5, supplementary Figure 2). At higher BOLD magnitudes, the SA increased considerably for Gaussian 1× and AWS, reaching values close to 100%. The trend was similar for the 2% noise level, which required BOLD magnitudes of 4% and higher to obtain optimal results. At the 4% noise level, only Gaussian 1.5× and AWSOM obtained an SA above 75%, requiring a BOLD magnitude of 6%. AWSOM performed remarkably well compared to other filters in preserving the SA of the small spheric ROA with decreasing BOLD magnitude and increasing noise levels.

Mask 2 had an irregular shape that was therefore challenging to preserve. It was the largest of all masks, with 903 voxels. For this mask, Gaussian 1×, medium SANLM, and AWS filters preserved the best SA (>80%) from a BOLD magnitude of 3% at noise levels of 1%. For BOLD magnitudes of 3% and 2% and noise levels of 4%, AWS maintained the best SA, along with AWSOM and Gaussian 2.5×.

Masks 3 and 4 have the same spheric shape and number of voxels, 257, but were positioned at different locations in the brain with distinct anatomical contrasts. The results were very similar between these two masks, where AWS was the best filter for preserving SA at all three noise levels from a BOLD magnitude of 2%. This was followed by AWSOM, and matched by Gaussian 2.5×, which preserved SA better with decreasing BOLD magnitudes and increasing noise levels. Evaluating the whole brain, AWS showed the best SA score overall, followed by AWSOM, and Gaussian 1× for higher BOLD magnitude and at lower noise levels.

### 3.5 Signal integrity

The SI metric was employed to quantitatively assess the underlying BOLD signal magnitude of activated voxels post‐filtering. This process entailed the calculation of correlations in spatial and magnitude dimensions between the ROA masks and the COPEs, which are thought to reflect the relative BOLD magnitude per voxel. This approach allowed us to assess the fidelity of the BOLD signal representation across ROAs after applying noise filters, providing insights into the effectiveness of each filtering technique in maintaining SI under varying conditions.

**FIGURE 6:**
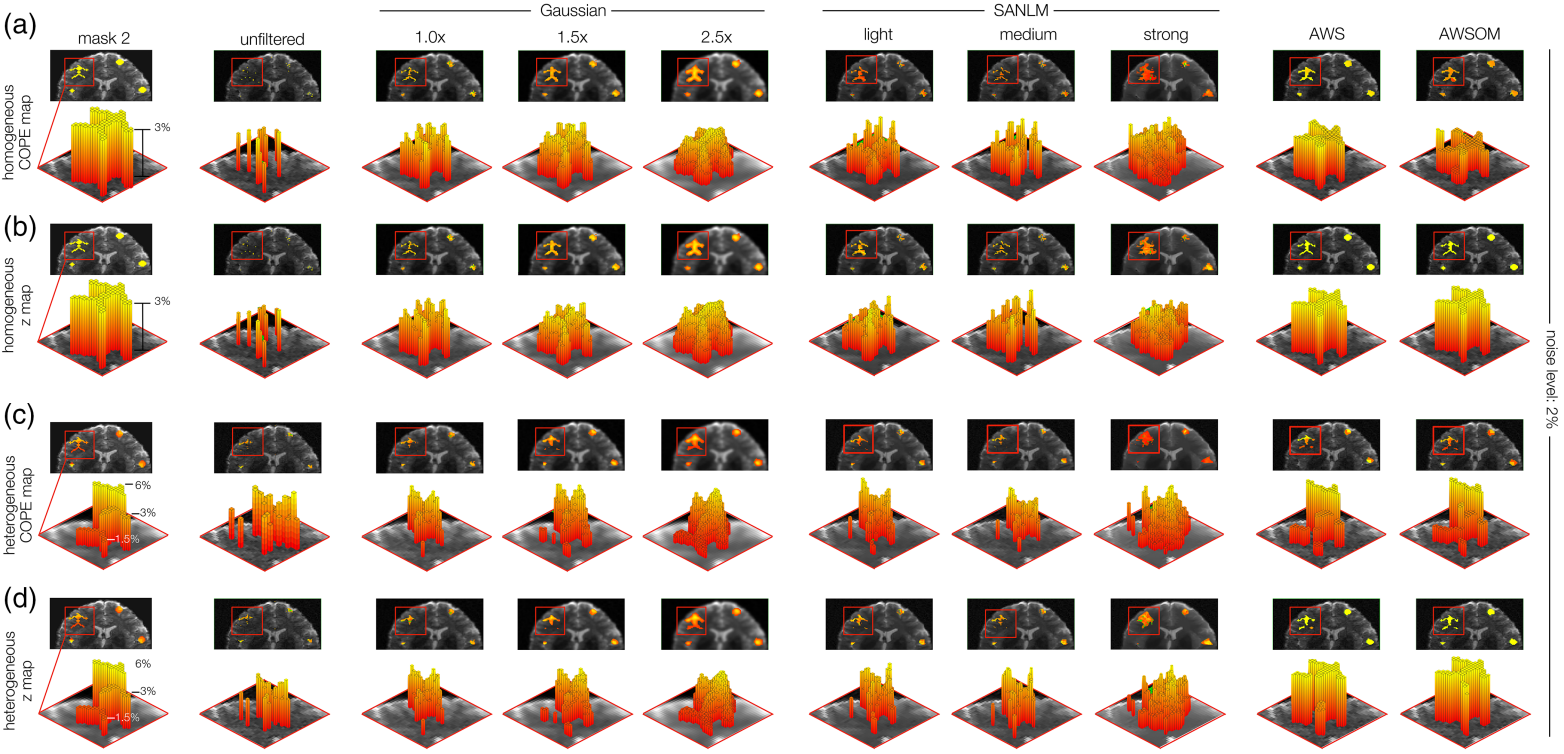
Comparison of the 3D BOLD magnitudes between the ground truth (here of mask 2) and the BOLD magnitudes obtained from the contrasts parameter estimates (COPEs) after applying the noise filters. (a) Homogeneous BOLD magnitudes at 3 and 2% of noise level (tSNR = 20). The first column shows the ground truth as a reference. This visualization allows us better to understand the filters' effects on the BOLD signal. The Gaussian filters have a more visible attenuation effect on the sides, while the SANLM filters mitigations do not follow a clear pattern. (b) Z‐maps of homogeneous BOLD activity at 2% of noise level. (c) COPEs of heterogeneous BOLD magnitudes (1.5, 3, and 6%) at 2% of noise level. (d) Z‐maps of heterogeneous BOLD activity at 2% of noise level. Utilizing the signal integrity metric, we quantitatively evaluate the underlying signal post‐filtering by calculating both spatial and magnitude correlations between the absolute truth masks and COPEs. The Z maps for AWS and AWSOME show identical values within the cluster, but the COPEs reveal their strong preservation of the reference shape. These 3D representations confirm that both AWS filters effectively minimize signal disruption during denoising.

To illustrate the logic behind the SI metric, the COPEs, which have been masked with the area of the thresholded z statistical map, to consider only significant voxels (z ≥ 3.1), were plotted in 3D and compared with the ROAs as a reference and ground truth (Figure 6). We used mask 2 (complex shaped ROA) and its respective activation cluster with 3% BOLD magnitude for each filter at a noise level of 2% (tSNR 20) to challenge the filters' performances and emphasize their idiosyncrasies.

Despite the homogeneous distribution of BOLD magnitudes across the ROA, all filters tend to suppress the signal unevenly, enforcing their characteristic shapes onto the SI landscape (Figure 6a). Gaussian filters, in particular, showed notable signal attenuation toward the peripheries. The shapes produced by the SANLM filters were irregular and lacked a clear pattern. AWS and AWSOM produced a COPE cluster that strongly resembled the ROA but with a slightly convex surface. AWSOM also displayed some voxel tiles that protruded relative to their neighbors despite the homogeneous magnitude distribution in the ROA.

We next determined the correlation coefficient between COPEs and z‐maps within significant voxel clusters, to estimate the extent to which the relative BOLD magnitude distribution was already reflected in the z‐scores (Supplementary Figures 8 and 9). This was interesting because z‐maps and not COPEs are conventionally used in fMRI. Barely differences between z‐scores and COPEs were found for Gaussian filters, as indicated by high correlation coefficients, especially at lower noise levels. For SANLM, we found random voxel peaks that differed for COPEs and z‐scores. AWS and AWSOM showed low correlation coefficients between COPEs and z‐scores. The ROAs with flat surfaces of homogeneous magnitudes were resembled when using z‐maps, but not for COPEs.

For ROAs comprising heterogeneous BOLD magnitude levels (Figure 6b), neither Gaussian nor SANLM filters could maintain the mask's original shape or magnitude distribution. In contrast, AWS and AWSOM preserved the mask shape and magnitude distribution with high fidelity for COPEs. The z‐maps of AWS‐based filters heavily flattened the integrity landscape, making differentiation of heterogeneous magnitude distributions impossible.

**FIGURE 7:**
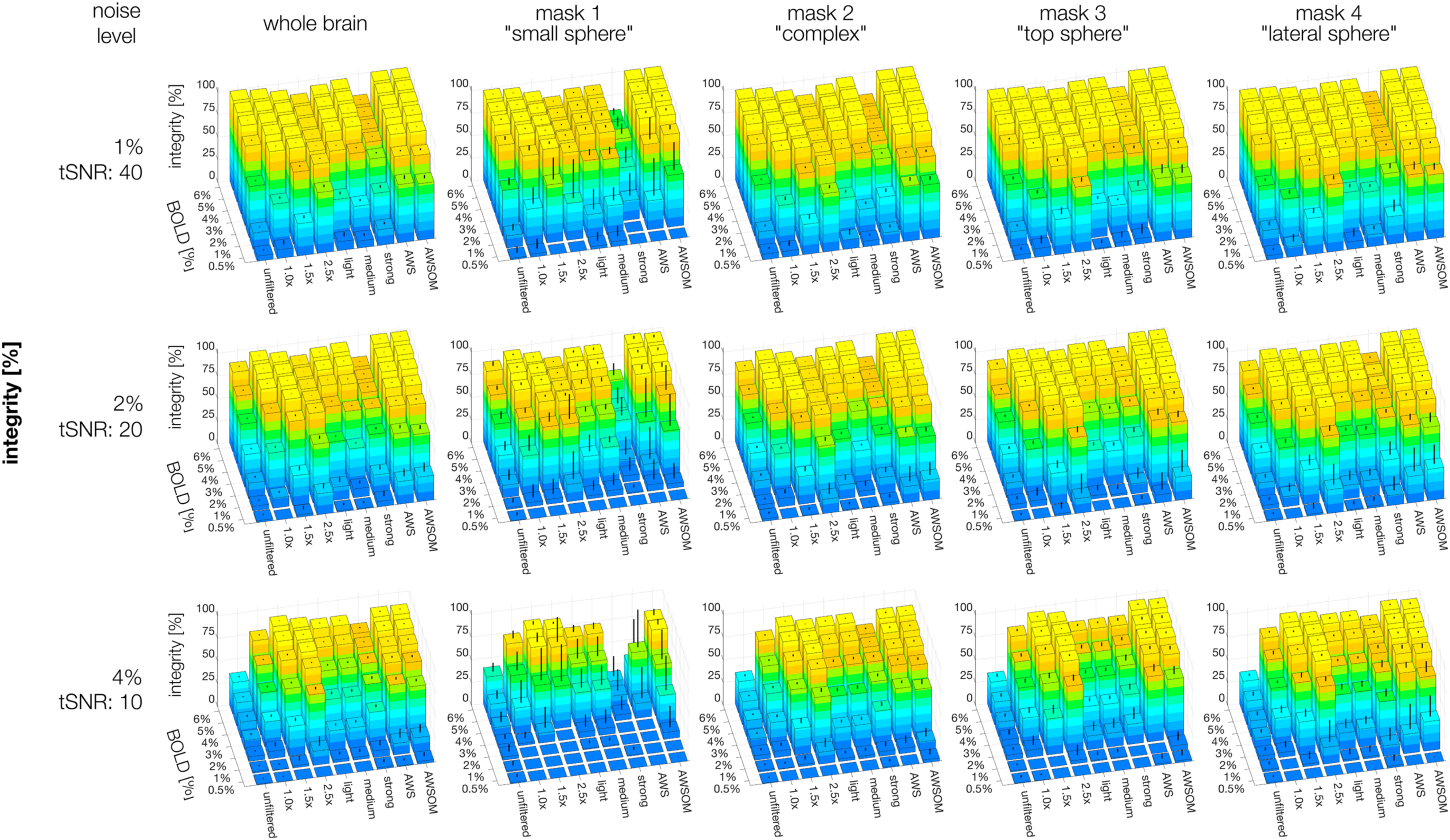
Signal integrity was measured on seven homogeneous BOLD magnitudes (0.5–6%) and three noise levels (1, 2, 4%) of Gaussian noise distribution. The error bars indicate the standard deviation. BOLD magnitudes of 0.5% were too small to be distinguished from noise, even after filtering, resulting in very low values for all filters. Overall, the AWSOME filter best preserves the integrity of the BOLD signal across the four masks and the whole brain.

In analyzing homogeneous ROAs across noise levels and BOLD magnitudes, AWS and AWSOM demonstrated superior SI at all noise levels across all masks (Figure 7, supplementary Figure 3). AWSOM performed particularly well for the small cluster mask (mask 1), which AWS could not detect properly. For BOLD magnitudes of 1% or higher, AWSOM consistently yielded SI values exceeding 70%. With AWSOM applied to mask 1 at a noise level of 4%, the method exhibited significant variability at smaller magnitudes (3% or less), as evidenced by the standard deviation indicated by the error bars in the figure.

### 3.6 Evaluation of SA, BS, and SI on heterogeneous data

**FIGURE 8:**
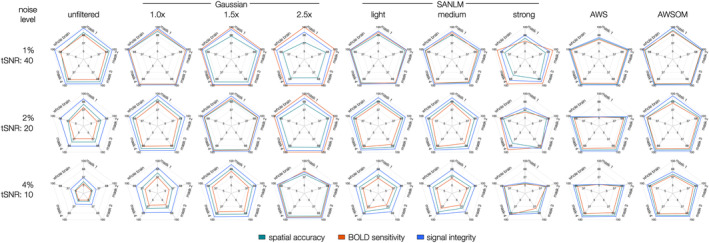
Spatial accuracy, BOLD sensitivity, and signal integrity were evaluated on simulated fMRI data sets using heterogeneous BOLD magnitudes (1.5–6%) and three noise levels (1, 2, and 4%) of Gaussian noise distribution. Each axis corresponds to the analysis performed, which are the separate masks and the whole brain. The plotted lines correspond to the metrics spatial accuracy, BOLD sensitivity and signal integrity. The Gaussian filters performed well as a function of kernel size and noise level. On the other hand, AWSOM performs equally well to the Gaussian filters regardless of noise level, being the best‐balanced filtering method. The 2.5× Gaussian filter obtained the highest sensitivity, although its accuracy was among the worst, especially at 1% noise, where it displayed low values. The AWSOM filter had the best and most balanced performance, including the very small patterns of ground truth mask 1, for which the standard AWS had the worst performance across the three metrics—revealing its deficiency in detecting small voxel clusters.

To identify high‐ and low‐performing filters in a near‐realistic scenario, we evaluated SA, BS, and SI for heterogeneous ROAs (with BOLD magnitudes of 6, 3, and 1.5%) across all masks and the entire brain at a tSNR of 40, 20, and 10. The results are summarized in spider plots (Figure 8).

AWS achieved the best stable and superior outcomes for BS, SA, and SI at 1 and 2% noise levels in the analysis of the entire brain, as well as masks 2, 3, and 4. This was tightly followed by AWSOM. Gaussian 1× performed especially well for high tSNR. At low tSNR of 10, Gaussian filters with 2.5× kernel size outperformed other filters, ranking as the most effective, with AWSOM following closely in second place.

In evaluating the very small spheric mask 1, the 1× Gaussian filter demonstrated equilibrium among high BS, SA, and SI at noise levels of 1 and 2%. At a higher noise level of 4%, the Gaussian filter with a 1.5× kernel size emerged as the most effective, although it registered SA and BS values under 65%. In contrast, the AWS filter exhibited the least favorable outcomes across all three metrics in mask 1. AWSOM, on the other hand, showed an optimal balance between the three metrics. The SANLM filters displayed intermediate performance, with the strong SANLM variant showing the least favorable results.

### 3.7 Filter evaluations in task‐based single‐subject fMRI data at 7 T

**FIGURE 9:**
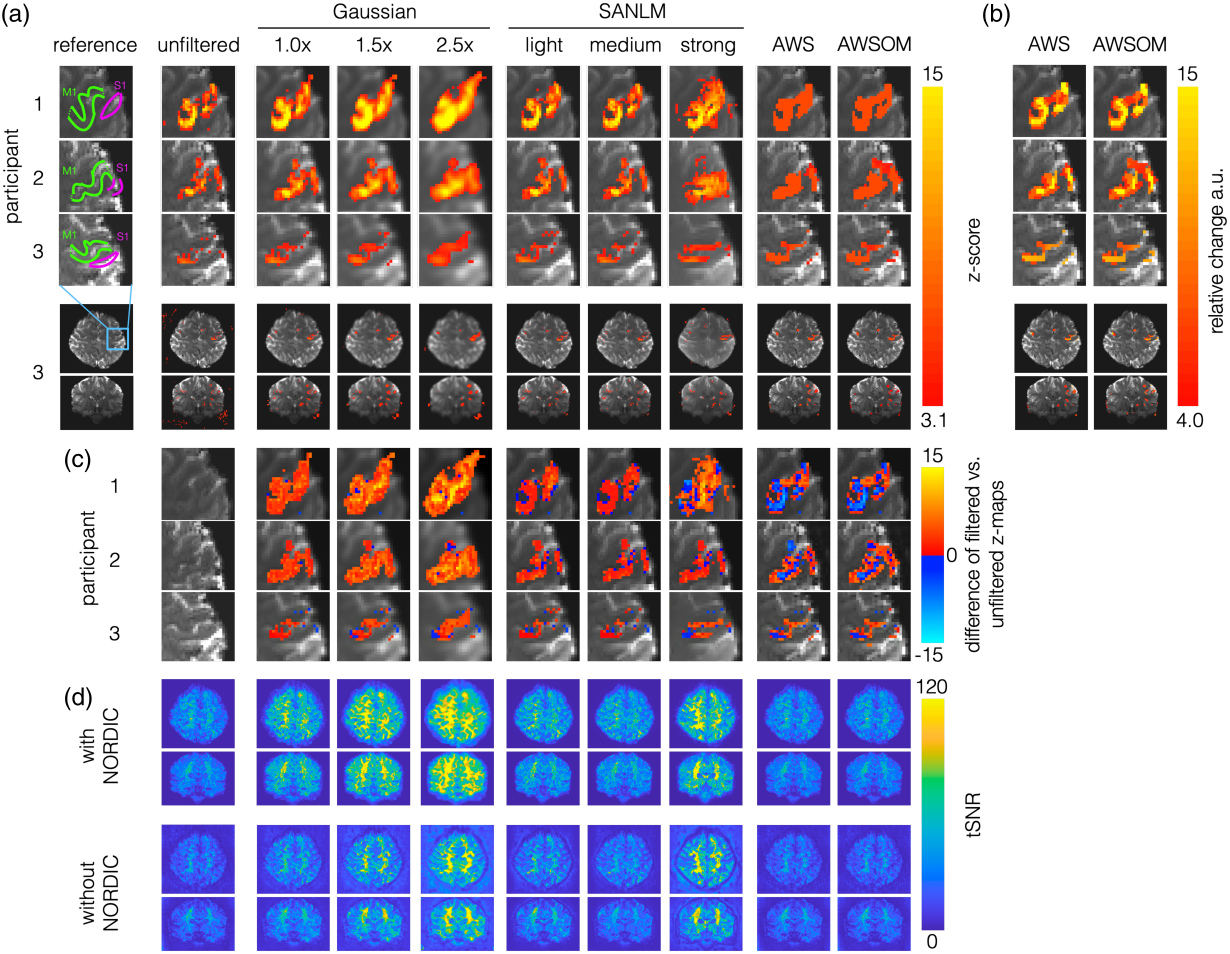
Comparison of the BOLD response in the primary motor cortex (M1) and primary somatosensory cortex (S1). A finger‐tapping experiment, right‐hand fingers against the thumb, was performed on three healthy participants. (a) The first column displays the reference for the expected regions of activation in M1 (green), S1 (pink). The subsequent columns present the resulting activity z maps obtained after applying each of the filtering techniques. The 2.5× Gaussian filter's smoothing effect resulted in an increased number of FP in neighboring regions and significantly blurred the image. SANLM light and medium intensities showed reasonable BOLD clusters that maintained the M1 boundaries. SANLM's strong intensity on participants 2 and 3 produces excessive FP. Both AWS filters revealed a thin continuous BOLD cluster along the M1 cortex in all three participants. The Z scores for AWS are lower, approximately 6, compared to other filters. However, these Z values are above the significance threshold (≥3.1), confirming their statistical significance. Fourth row: Axial view of the entire brain of participant 3. Fifth row: Coronal view of the entire brain. Displaying the whole brain allows for a comprehensive visualization of all activity within the brain and, in certain instances, activity detected outside the brain, as observed with Gaussian 1.0×, SANLM strong, and unfiltered data. (b) COPE maps masked with z maps. By utilizing both Z maps and COPEs, we can ascertain which voxels are statistically significant and comprehend the magnitudes of BOLD activity. These maps illustrate areas of stronger brain activity. The COPEs maps use arbitrary units that are based on the relative change from the baseline. (c) The difference between each filter and the unfiltered results in panel (a) are shown. The unfiltered after NORDIC denoising is considered as a reference to show the variations in the activation produced by each filter. (d) Temporal signal‐to‐noise ratio (tSNR) maps. These maps present the spatial distribution of tSNR in axial and coronal view, using the same slices as in (a) of participant 3. For reference, a comparison of the tSNR obtained before and after applying the NORDIC denoising is presented.

We next conducted a feasibility study on three volunteers to test the filter performances in task‐based fMRI data. The study involved a comparative analysis of the BOLD response in the primary motor cortex (M1) during a finger‐tapping task, where participants tapped their right‐hand fingers against the thumb (Figure 9). The data were acquired with an isotropic voxel size of 1.5 mm and denoised using NORDIC (Vizioli et al., 2021).

Gaussian filters with 1×, 1.5×, and 2.5× kernel sizes displayed a considerable number of significant voxels in regions neighboring M1 (Figure 9a,b) and beyond the brain's confines (Figure 9c). The SANLM filters at light and medium intensities yielded more accurate BOLD clusters, effectively preserving the boundaries of the M1 region (Figure 9a). The strong SANLM filter resulted in substantial deviations of the BOLD cluster's shape in participants 1 and 2 compared with the cluster shapes preserved by all other filters. AWS and AWSOM delineated a slender, continuous BOLD cluster along the M1 cortex in all three participants. The differences in the z‐scores for each filter compared to that of unfiltered data are presented to facilitate the comparison of the effects of the applied smoothing filters (Figure 9c). The unfiltered data led to detect some voxels that are potentially FPs; these are indicated as negative values in the difference maps of the smoothed data. For SANLM and AWS‐based filters, the negative clusters along M1 occur because their z‐values are lower than those of the unfiltered data. As observed in Figure 6, the z maps of AWS‐based filters flattened out all gradual differences in BOLD magnitudes. We, therefore, replaced significant voxels with COPEs to display the SI (Figure 9b). The gradations exhibited coarser increments compared to those of the Gauss and SANLM filters. AWS‐based filters, and SANLM light and medium, displayed their maxima without introducing additional gradations tapering around them, as seen with all Gaussian filters.

In addition to the clusters in the contralateral M1, other BOLD effects in the somatosensory‐interoceptive‐motor pathway—such as the contralateral primary somatosensory cortex (S1), thalamus, posterior insula, cingulate cortex, and ipsilateral M1—that were vaguely discernible through other filters were further accentuated by AWS and AWSOM, as exhibited in the last files in panels a and b of Figure 9. tSNR maps are displayed to indicate variability across brain regions before and after applying NORDIC denoising (Figure 9d).

### 3.8 Evaluating FPR and distribution in single‐subject resting‐state fMRI at 7 T

**FIGURE 10:**
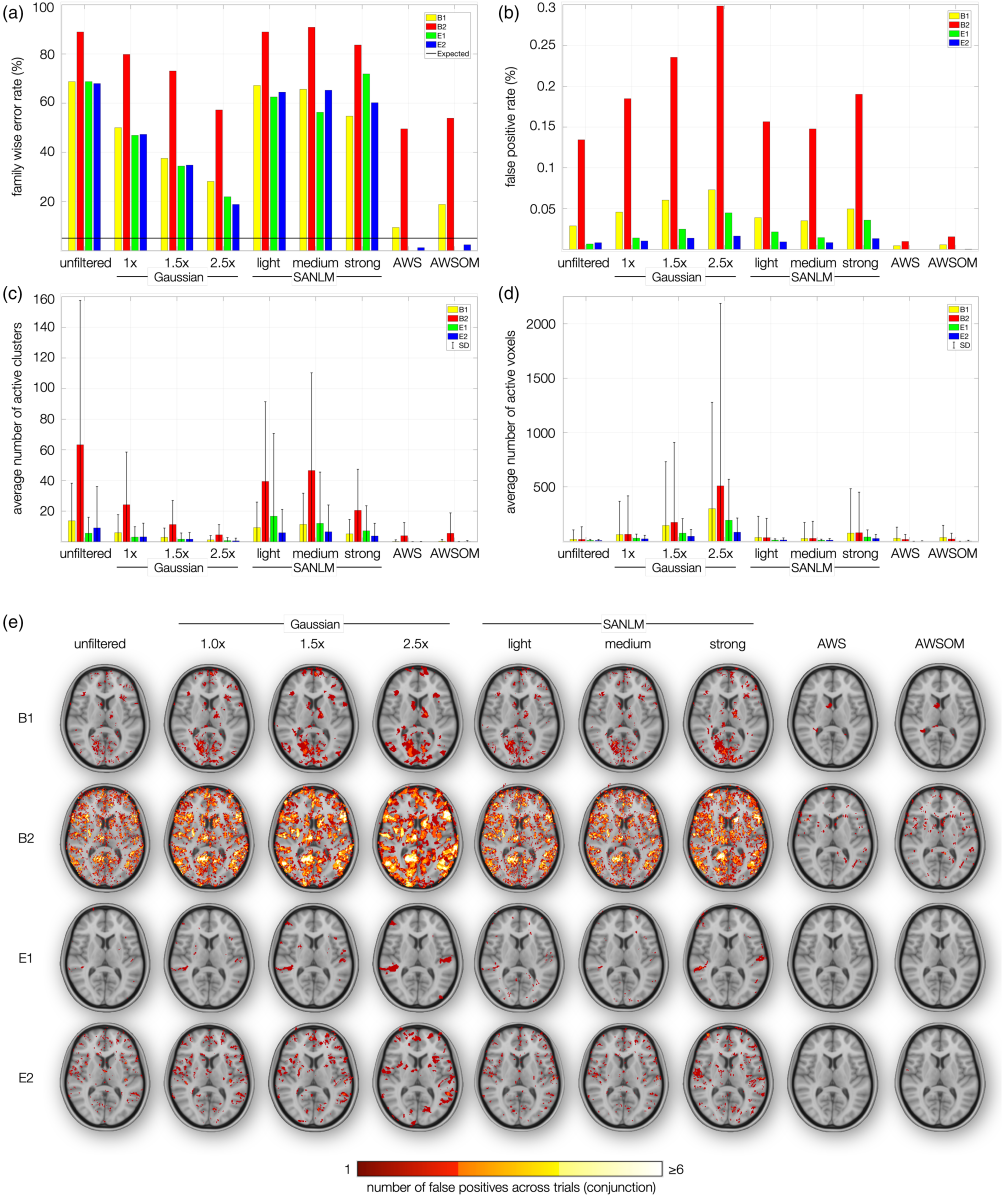
Analysis of family‐wise error rate (FWER) and false positive rates in fMRI data. (a) FWER, following the methodology established by Eklund et al. (2016). Two block paradigms (B1, B2) and two event related paradigms (E1, E2) were used on resting‐state fMRI time series, presuming cero activity, therefore, all active voxels are false positives. B1 consisted in 10 s on and 10 s off (10 on/off). B2: 30 s on/off. E1: 2 s active and 6 s' rest. E2: Active between 1 and 4 s, and 3–6 s rest, pseudorandomized. The nominal FWER of 5% is indicated with a black line. The SANLM filters displayed the highest FWER. (b) The false positive rate was quantified by the ratio of falsely activated voxels to the total voxel count within the brain. Despite the elevated FWER depicted in (a), (b) reveals that the proportion of falsely activated voxels did not exceed 0.3% of the total brain volume. This panel further elucidates the impact of Gaussian smoothing on the inflation of false positives, in contrast to AWS filters, which significantly mitigate such false activations. Notably, AWS filters achieved a zero false positive rate for the E1 paradigm and maintained rates near zero for E2, underscoring their efficacy in enhancing data fidelity. (c) Average number of active clusters. (d) Average number of active voxels. (e) Distribution of false positives. The maps in panel (e) provide insight into the spatial distribution and extent of activation.

In the previous sections, we evaluated the advantages and disadvantages of each filter on synthetic fMRI data. We then tested the filters for experimental task‐based 7 T fMRI results in the individual brain, observing significant voxels that may or may not be related to the applied task. To assess whether the applied filters produce inflated FPRs in single‐subject analysis for 7 T fMRI data under the assumption that the null‐hypothesis is true, we employed the tests proposed by Eklund et al. (2016). These tests evaluated FP results across two block (B1, B2) and two event‐related paradigms (E1, E2) on resting‐state data (Figure 10).

Block paradigm B1 presented interleaving stimulus and resting periods of 10 s each, B2 of 30 s. For event‐related paradigm E1, we modeled a stimulus duration of 2 s, followed by 6 s of rest; E2 had varying stimulus periods of 1–4 s, interleaved by 4–6 s rest. We ascertained the empirical FWER for each condition and filter, which reflects the percentage of trials showing at least one FP voxel (Figure 10a). This was compared against the FPR, comprising the ratio of falsely activated voxels to the total voxel count within the brain (Figure 10b). These observations were contextualized with the average number of FP clusters (Figure 10c) and voxels per condition and filter (Figure 10d).

The FWERs for the tested filters in single subjects were remarkably high. Excluding block paradigm B2, the FWER averaged around 70% for unfiltered data, decreasing to around 50% for Gaussian 1×, approximately 35% for Gaussian 1.5×, and between 20 and 30% for Gaussian 2.5×, depending on the paradigm. Therefore, for Gaussian filters, the reduction in FWER can be read as a function of the smoothing kernel size. The SANLM filters presented the highest percentage of FWER, approaching the values obtained by the unfiltered for B1, E1, and E2, and even surpassing the values for B2. AWS and AWSOM produced substantially lower FWER than other filters, falling below the nominal 5% rate for the event‐related paradigms E1 and E2. FWER tended to generally increase with the duration of a paradigm's on–off periods, with event‐related E1 and E2 producing lower FWER than the block paradigm B1. B2 produced strikingly high FWER for all methods, with about 90% for unfiltered data, 80, 72, and 58% for Gaussian filters 1×, 1.5×, and 2.5×, respectively, 50% for AWS, and 55% for AWSOM (Figure 10a).

Although the FWER was high, the FPR showed that the identified activations constituted less than 3% of the total brain volume for all the filters and paradigms (Figure 10b). While FWER decreased with the extent of smoothing, for FPR this relationship was reversed, with larger smoothing kernels FPR ranging for block‐paradigm B2 from 0.17 for unfiltered data to 0.3 for Gaussian 2.5×. The FPR of the SANLM filters were less than 0.05% for all paradigm types, except for B2, where the ranges were less than 0.2%. Other paradigms produced substantially lower FPR, ranging from 0.01 to 0.07. AWS and AWSOM obtained the lowest FPR across all filters.

Increasing the kernel size of the Gaussian filter reduced the average number of clusters (Figure 10c), but increased the number of active voxels (Figure 10d). The Gaussian filter at 2.5× produced the largest number of active voxels with a considerably large standard deviation. AWS and AWSOM maintained the average number of clusters and voxels lower than all other filters, showing their superiority in suppressing FPs. Figure 10e illustrates the distribution of FPs in the brain with a conjunction map across all trials per filter and paradigm. The B2 paradigm shows active voxel clusters distributed throughout the brain, heavily adding up across multiple trials for all three Gaussian filters, and SANLM in its three intensities. AWS and AWSOM filters exhibited tremendously fewer FPs that barely occurred at the same position for two trials.

## 4 DISCUSSION

In this work, we developed a framework to examine the efficacy of different smoothing and inference methods for high spatial resolution, single‐subject fMRI at 7 T. The overall goal and achievement of our work is to enhance BS while preserving SA and SI of identified BOLD clusters across the brain under the premise of keeping FP activations in check. Our main finding is that AWSOM best balanced the demands of high spatial resolution fMRI for all noise levels in the brain and overcame the three key challenges of single‐subject fMRI at high spatial resolution. The first challenge lies in enhancing the BS for large and small clusters while preserving the SA over a considerable range of tSNR. The second challenge focuses on SI—in other words, a filter's effect on the relative BOLD magnitudes of activated voxels within a detected cluster. The third challenge concerns our overall capacity to distinguish signal from noise while keeping the likelihood of producing FPs to a minimum to ensure that the inferences made from the analysis are correct.

To address the first challenge, we aimed to improve BS while maintaining SA across the varying tSNR present throughout the brain. Large Gaussian filters strongly increased BS but came with a loss of SA. This could be improved using small Gaussian kernels (1×, 1.5×) at higher tSNR. The lower the tSNR and BOLD magnitude, the more Gaussian smoothing was necessary to balance sensitivity and accuracy. tSNR can vary drastically depending on the type of study, especially when the exact location and size of the expected activations are not known (Colizoli et al., 2020). The problem with this trivia is the inhomogeneous sensitivity profile of the radio frequency (RF) head array used for signal reception (Avdievich et al., 2009). The tSNR in the cortex—which is close to the receive elements of the RF head array—is approximately twice as high as in subcortical regions such as the thalamus, whereas the tSNR of deeper regions such as the brainstem easily drops to about one‐third (Colizoli et al., 2020; Miletić et al., 2020; Vizioli et al., 2021). This means that a Gaussian filter of 1×, which perfectly enhances both BS and SA in the cortex, may already lose weaker BOLD signals in the thalamus and might not detect anything in the brainstem. Using large Gaussian kernels to significantly enhance subcortical BOLD effects comes at the expense of SA of cortical BOLD patterns at high tSNR. Attempts to improve tSNR based on thermal denoising and increasing magnetic field strengths have been suggested to render smoothing obsolete (Vizioli et al., 2021). This might be true when examining high‐magnitude BOLD effects in cortical regions with a tSNR well above 40. Yet, this is not advisable for lower tSNR or whole‐brain examinations. In this regard, AWSOM proved itself a commendable asset, exhibiting strong BS and SA across all noise and BOLD magnitude levels that were tested. Its sensitivity to smaller clusters represents the initial improvement over AWS.

To tackle the second key challenge, we introduced SI as a new metric. With the advent of decoding functional neurosignatures, the research questions shifted from *which* areas are activated to *how* areas are activated (Caballero‐Gaudes & Reynolds, 2017; Ekstrom, 2010; Logothetis & Pfeuffer, 2004). Thus, assessing the relative BOLD magnitudes of activated voxels becomes highly relevant. Since significance has generally played a major role in fMRI, little attention has been paid to the effects of smoothing on the BOLD signal magnitudes. To investigate this adverse effect, we used the COPE maps—which reflect the magnitude of the BOLD signal when contrasted with the baseline or resting period (Smith, 2004; Smith et al., 2007)—and masked them with the z‐maps to ensure only statistically significant voxels were considered. Gaussian filters imprinted a characteristic bell‐shaped curve on the BOLD clusters that were detected, with central voxels exhibiting high relative activities that decay toward the periphery. This effect intensified with increasing kernel size. In contrast, AWS and AWSOM algorithms do not involve any global smoothing of the time series, but target the COPE maps, where smoothing is conducted locally depending on the relative BOLD magnitudes and the variances of neighboring voxels (Polzehl et al., 2010; Polzehl et al., 2020; Tabelow et al., 2006; Tabelow et al., 2011). SI complemented our precision analysis by assessing the preservation of the relative magnitudes of the BOLD signal. AWS‐based filters showed superior signal handling by preserving the relative BOLD magnitudes across the significant voxels.

The third challenge was addressed by employing the null‐hypothesis test proposed by Eklund et al. (2016). This challenge is particularly relevant for high‐resolution single‐subject fMRI, as FPs are not averaged out, unlike in group analyses. Furthermore, it was shown that the FWER substantially increases with higher resolution (Mueller et al., 2017). We modeled various task paradigms on resting‐state data, defining every significant voxel as a FP. The goal was to estimate FWER, the chance of one or more FPs anywhere in the brain, and FPR, the percentage of FP voxels relative to the brain volume. AWS and AWSOM outperformed all other methods regarding FWER and FPR. When we searched for event‐related paradigm‐like signal changes with brief on–off periods in resting‐state fMRI data, we found that AWS and AWSOM were the only methods that kept FWER in single subjects below the expected nominal value of 5%. Thus, they posed no significant risk of detecting FPs, as assessed by the procedure proposed by Eklund et al. (2016). AWS‐based methods smooth the statistical landscape locally based on the beta contrasts and variances of neighboring voxels, flattening noise, and emphasizing activated clusters (Tabelow et al., 2006; Tabelow et al., 2009; Tabelow et al., 2011; Tabelow & Polzehl, 2011). AWS still estimates a global statistic threshold based on the data's smoothness; but due to the amplified differences of detected signals versus noise the exact thresholding is less critical compared to more conventional methods in fMRI, including cluster correction. Conventional inferences rely on exact estimation of smoothness to determine the statistical significance of voxel clusters, which requires Gaussian smoothing (Tabelow et al., 2006; Tabelow et al., 2009; Tabelow & Polzehl, 2011). In theory, Gaussian kernels of at least double the voxel size are required to overwrite the intrinsic correlation structure of acquired data and determine significance (Poldrack et al., 2011). Our data provides experimental insights into FWER of single subjects across several Gaussian kernel sizes, including voxel‐sized kernels to improve SA. Smaller kernels have a higher probability of detecting at least one FP per trial (higher FWER) but show fewer FPs per trial (lower FPR) than larger kernel sizes. In practice, failure to respect the FWE can have various complex causes that stem from a more complicated local correlation structure across voxels or deviations from distributional assumptions (Eklund et al., 2016; Kriegeskorte et al., 2008; Noble et al., 2020). This highlights the importance of assessing the FWER from acquired data over synthetic datasets. The high FWER and FPR across all filters, except AWS and AWSOM, illustrate the inflated cluster correction failure in high‐resolution single‐subject fMRI.

Remarkably, we found dramatically heightened FWER and FPR for modeling block paradigms with longer on–off periods in the resting‐state data. The extremely high FWER across all filters for B2, with long on–off periods of 30 s, indicates that low‐frequency periodic signal changes have been modeled in the time series of multiple subjects. Low‐frequency oscillations can be caused by scanner‐related artifacts (Jamil et al., 2021; Vizioli et al., 2020; Vizioli et al., 2021), aliasing frequencies of periodic physiological signals such as respiration or heartbeat (Hutton et al., 2011; Triantafyllou et al., 2005; Van Der Zwaag et al., 2015). Another cause may be actual neurovascular activities such as functional networks emerging in the resting state (Caballero‐Gaudes & Reynolds, 2017; Vigotsky et al., 2020). The possibility of modeling slow‐oscillatory functional networks in a resting state distinguishes an approach like the one introduced by Eklund and colleagues from real task‐based experiments, as the functional network landscape adapts to the task and does not run independently in parallel (Cole et al., 2021). However, the block paradigms that were used are prone to lead to an inflated FWER for single subjects, which would be averaged out for group analysis.

The performance of individual filters in preventing FPs was particularly evident in the FPR. Here, AWS and AWSOM were extremely strict, with a near‐zero FPR for B2 and even lower rates for B1 and event‐related paradigms. FPRs of other methods were substantially elevated and skyrocketed for B2, increasing with Gaussian smoothing kernel size. Our assessments demonstrate how Gaussian kernel size affects FWER and FPR of high‐tSNR fMRI data in the individual brain. We strongly advise against using large smoothing kernels in single‐subject fMRI with high tSNR for the sake of keeping the FPR at bay. Depending on the research question, it may be preferable to interpret single false‐positive voxels scattering of unfiltered data than large artificial blobs produced by Gaussian smoothing. Nonetheless, it is essential to distinguish FPs from true activations (Desco et al., 2001; Noble et al., 2020). The AWS‐based filters offer remarkable reliability in this regard.

The SANLM filters warrant a brief summary in their own right. They yield the highest FWER across all filters for all paradigms and a remarkable FPR, both in the range of unfiltered data. This can be explained by the non‐Gaussian correlation structure of the data that led to failure in determining significance using cluster correction (Bansal & Peterson, 2018; Woo et al., 2014). Strong SANLM filtering, designed to remove high amounts of noise from submillimeter structural data, performs poorly in terms of SA and SI and only became interesting at extremely high noise levels. At a tSNR as low as 2.5, strong SANLM detected complex activation structures as the only filters, albeit in the form of amorphous blobs, as well as large spherical clusters also identified by AWS‐based filters. At high tSNR, medium SANLM filters perform roughly in the mid‐range between unfiltered and Gaussian 1× in SA and BS, showing impressively detailed graduations of cortical activations in finger tapping. However, it becomes evident how the shape of activation clusters fray toward subcortical regions, while Gaussian and AWS‐based filters represent thalamic nuclei as rounded structures. This effect may be expected to be pronounced with other data, as our example fMRI scan exhibited unusually homogeneous tSNR across the coronal section, which is uncommon in the literature (Colizoli et al., 2020; Miletić et al., 2020; Vizioli et al., 2021). The usage of SANLM filters in fMRI is fundamentally problematic because their spatial smoothing properties are guided by anatomical contrasts of the time series (Gaser et al., 2022; Manjón et al., 2010), potentially causing significant voxels to conform in their shape to the anatomy. Moreover, the resulting non‐Gaussian smoothing violates the criteria of Gaussian random field theory (Flandin & Friston, 2019; Hayasaka & Nichols, 2003; Worsley, 2003). Principally, SANLM filtering could be applied in the temporal dimension, raising the question of how to determine statistical inference. We included the current versions of SANLM (Gaser et al., 2022) in our tests due to previous application of SANLM filtering in the fMRI (Bernier et al., 2014) and their fidelity and gradations of cortical clusters at high tSNR levels for medium SANLM.

AWSOM exhibits a somewhat stepped graduation of BOLD magnitudes for experimental data. We recognize the limitation that we tested SI only for three steps of magnitudes and did not take finer graduations into account. Yet, our findings are a testament to the overall superiority of AWSOM over the other filters that we have tested. Our acquired data examples cover the motor cortex during finger tapping and do not involve a task that specifically engages subcortical areas. Using data from the *Human Connectome Project* (Marcus et al., 2013; Van Essen et al., 2012), which covers 7 T fMRI data in emotion regulation tasks, provides a valuable source and use case for analyzing subcortical areas. This would allow the use of subtle regions, such as the amygdala or nucleus accumbens, as region‐of‐interest for precise comparison. We did not evaluate the performance of AWSOM for extremely high tSNR, where voxel‐based inference methods may suffice to capture BOLD effects, and smoothing may not be necessary (Vizioli et al., 2021). Nevertheless, such high tSNR is typically assessed in the cortex and decreases toward deeper brain structures. Generally, extremely high tSNR will be translated into voxel size reduction and enhanced spatial resolution in exchange for tSNR reduction. Also, weak BOLD magnitudes may well be lost without any amplification. Finally, AWSOM sets a threshold for significance, which always comes at the risk of losing signals that cannot be distinguished from noise; they may be present, although insignificant, across many subjects. This is a general problem for single subject analysis. It will therefore be intriguing to look below the threshold. Yet, it will be essential to distinguish signal from noise. AWSOM has proven its ability to prevent type I errors by effectively suppressing false‐positives, while achieving a high sensitivity for the BOLD effect, thus minimizing type II errors.

## 5 CONCLUSION

To support precision fMRI at single‐subject level we introduced AWSOM, to balance the demands on the quality precision metrics that were tested for a large range of noise levels. Using small Gaussian smoothing kernels is a viable option since their performance can be very accurate depending on the noise level. However, it is crucial to consider both the noise level and the expected activation size to ensure optimal performance. Furthermore, Gaussian filters are generally prone to detect FP activations in the brains of single subjects at high spatial resolutions. AWSOM offers a one‐size‐fits‐all solution to detect BOLD signals of varying magnitudes across the wide range of tSNR throughout the brain with high BS, SA, and SI. AWSOM allows for a balance between sensitivity to weak BOLD signals and the suppression of FPs, thus effectively managing the trade‐off between type I (false‐positives) and type II errors (false‐negatives). AWSOM is being made available to the community.

## FUNDING INFORMATION

This project has received funding from the European Research Council (ERC) under the European Union's Horizon 2020 research and innovation program under grant agreement No 743077 (ThermalMR).

## CONFLICT OF INTEREST STATEMENT

The authors declare that there is no conflict of interest.

## Supporting information


**SUPPLEMENTARY FIGURE 1.** Sensitivity evaluated on simulated fMRI data sets using 7 homogeneous BOLD magnitudes (0.5–6%) and three noise levels (1, 2, 4%) with Gaussian noise distribution. The average of 10 simulations was calculated for each case. The values in these heat maps correspond to the results shown in the main Figure 4.

**SUPPLEMENTARY FIGURE 2.** Accuracy evaluated on simulated fMRI data sets using 7 homogeneous BOLD magnitudes (0.5–6%) and three noise levels (1, 2, 4%) with Gaussian noise distribution. The average of 10 simulations was calculated for each case. The values in these heat maps correspond to the results shown in the main Figure 5.

**SUPPLEMENTARY FIGURE 3.** Signal integrity was measured on 7 homogeneous BOLD magnitudes (0.5–6%) and three noise levels (1, 2, 4%) with Gaussian noise distribution. The average of 10 simulations was calculated for each case. The values in these heat maps correspond to the results shown in the main Figure 7.

**SUPPLEMENTARY FIGURE 4.** Sensitivity evaluated on simulated fMRI data sets using 7 homogeneous BOLD magnitudes (0.5–6%) and three noise levels (1, 2, 4%) with Rician noise distribution. The performance of all filters is comparably high at 1 and 2% noise levels.

**SUPPLEMENTARY FIGURE 5.** Accuracy evaluated on simulated fMRI data sets using 7 homogeneous BOLD magnitudes (0.5–6%) and three noise levels (1, 2, 4%) with Rician noise distribution. The average of 10 simulations was calculated for each case. AWS and AWSOM show dominance in each of the conditions, except in the small cluster (mask 1), where Gaussian 1.5x performed best, followed by AWSOM.

**SUPPLEMENTARY FIGURE 6.** Signal integrity was measured on 7 homogeneous BOLD magnitudes (0.5–6%) and three noise levels (1, 2, 4%) with Rician noise distribution. BOLD magnitudes of 0.5% were too small to be distinguished from noise, even after filtering, resulting in very low values for all filters. AWSOM was superior in preserving the integrity of the BOLD signal.

**SUPPLEMENTARY FIGURE 7.** Spatial accuracy, BOLD sensitivity, and signal integrity were evaluated on simulated fMRI data sets using heterogeneous BOLD magnitudes (1.5–6%) and three noise levels (1%, 2%, and 4%) with Rician noise distribution. Each axis corresponds to the analysis performed, which are the separate masks and the whole brain. The plotted lines correspond to the metrics spatial accuracy, BOLD sensitivity and signal integrity. As in the simulations with Gaussian noise distribution, AWSOM was the most balanced for all noise levels.

**SUPPLEMENTARY FIGURE 8.** **(A)** Correlation coefficient heatmaps between Contrast Parameter Estimates (COPE) maps and z maps for homogeneous time series with Gaussian distributed noise. **(B)** Correlation coefficient between COPE maps and z maps for homogeneous time series with Rician distributed noise. The correlation coefficients were multiplied by a 100 to display the results in percentage. Gaussian filters displayed a nearly perfect correlation in low noise conditions and high BOLD magnitudes. On the other hand, AWS methods depict an extreme low correlation showing that its algorithm handles these parametric maps differently and that they cannot be used interchangeably.

**SUPPLEMENTARY FIGURE 9.** **(A)** Correlation coefficient heatmaps between COPE maps and z maps for heterogeneous time series with Gaussian distributed noise. **(B)** Correlation coefficient between COPE maps and z maps for heterogeneous time series with Rician distributed noise. The correlation coefficients were multiplied by a 100 to display the results in percentage. Again, Gaussian filters had a high correlation between maps. The SANLM filters had an intermediate level of correlation, with the strong intensity having the lowest correlation. AWS and AWSOM reached the lowest values, indicating almost zero correlation.

## Data Availability

We are currently compiling all necessary scripts to make AWSOM available for download. The task‐based and resting‐state fMRI data can be shared upon request.
