# Supplementary figures and images for: Precision fMRI and cluster‐failure in the individual brain
Source: Hum Brain Mapp. 2024 Aug 26;45(12):e26813. doi: 10.1002/hbm.26813 (PMC11345700; doi:10.1002/hbm.26813)

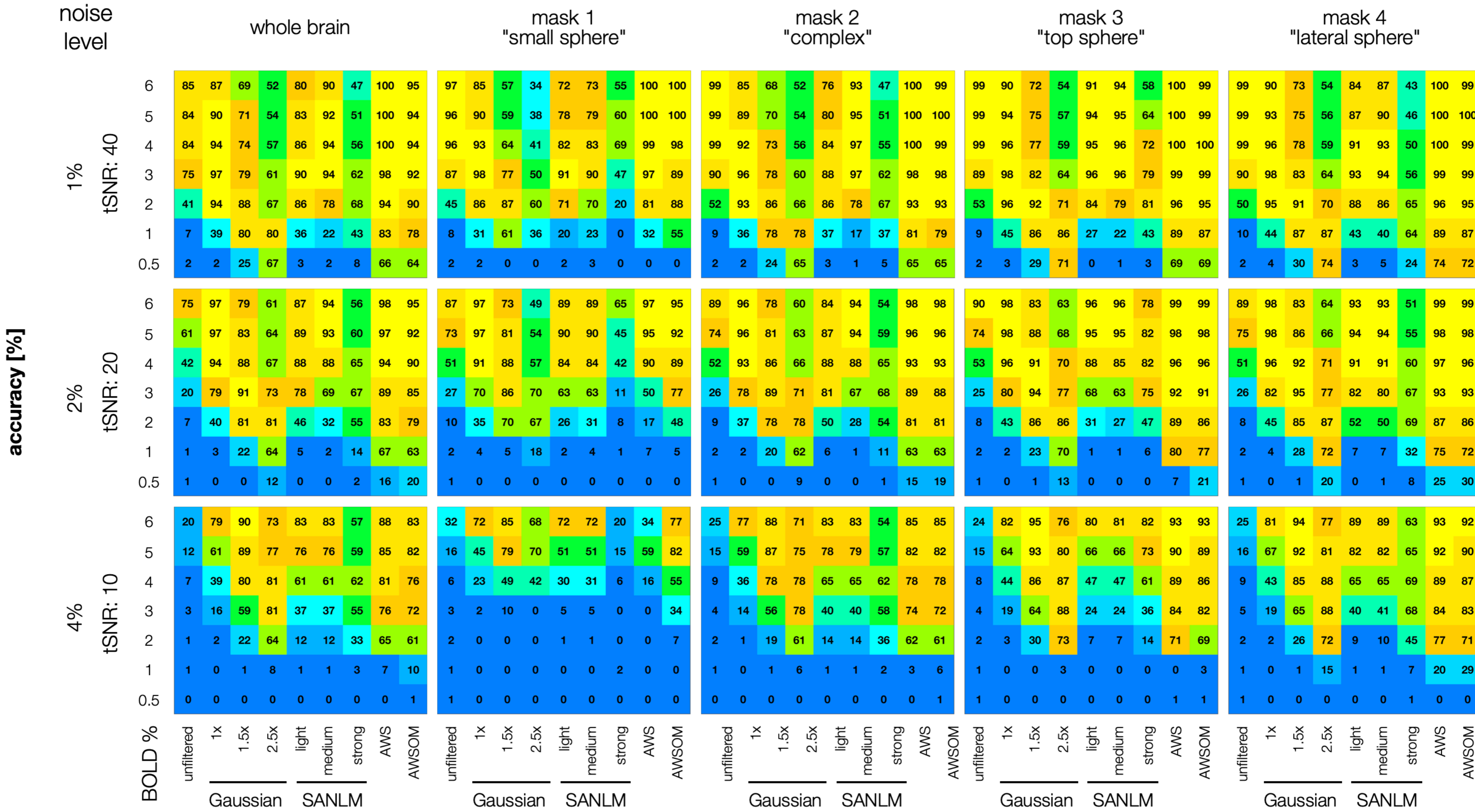

Supplement: Supplementary file 2 — SUPPLEMENTARY FIGURE 2. Accuracy evaluated on simulated fMRI data sets using 7 homogeneous BOLD magnitudes (0.5–6%) and three noise levels (1, 2, 4%) with Gaussian noise distribution. The average of 10 simulations was calculated for each case. The values in these heat maps correspond to the results shown in the main Figure 5. [file HBM-45-e26813-s004.pdf]

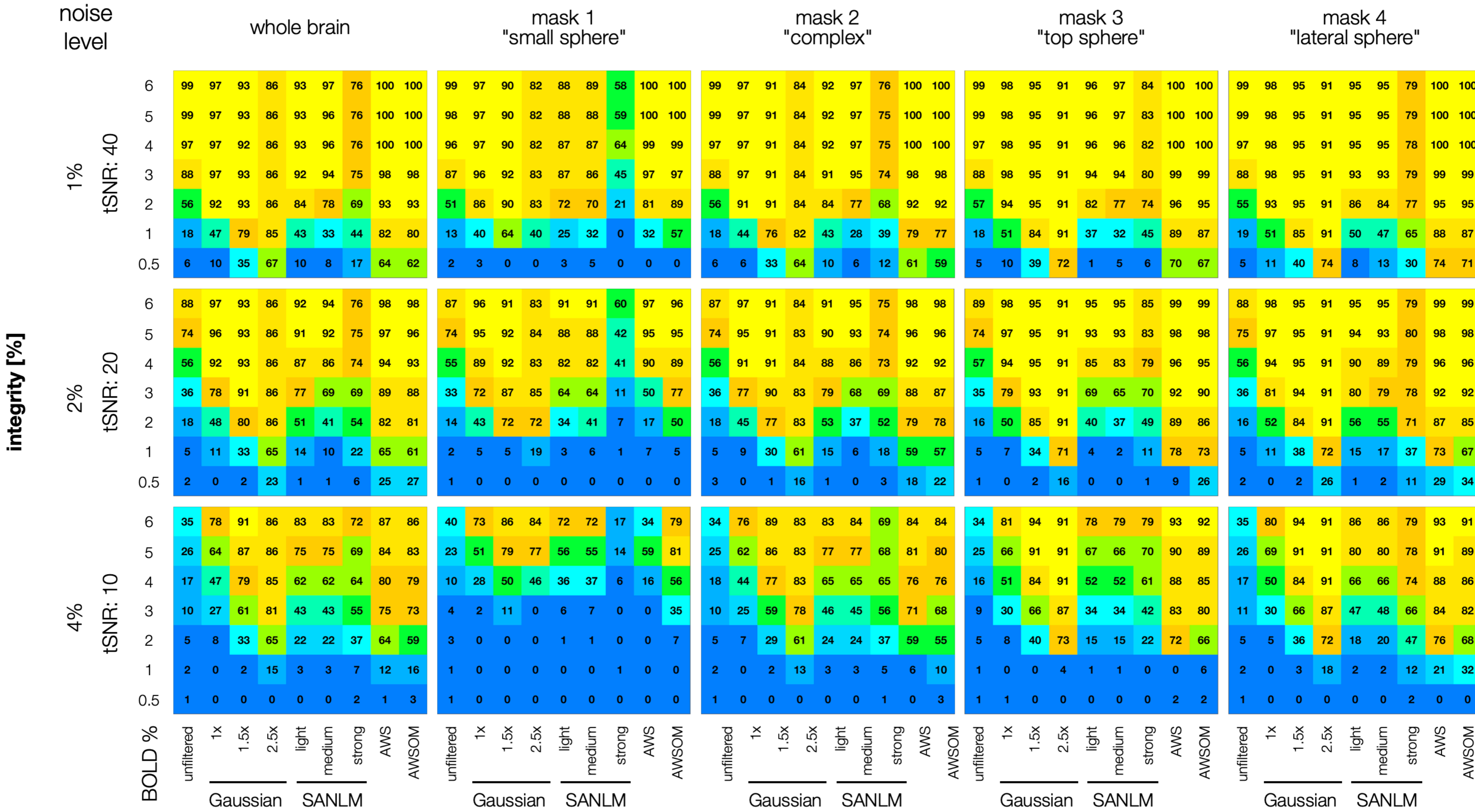

Supplement: Supplementary file 3 — SUPPLEMENTARY FIGURE 3. Signal integrity was measured on 7 homogeneous BOLD magnitudes (0.5–6%) and three noise levels (1, 2, 4%) with Gaussian noise distribution. The average of 10 simulations was calculated for each case. The values in these heat maps correspond to the results shown in the main Figure 7. [file HBM-45-e26813-s001.pdf]

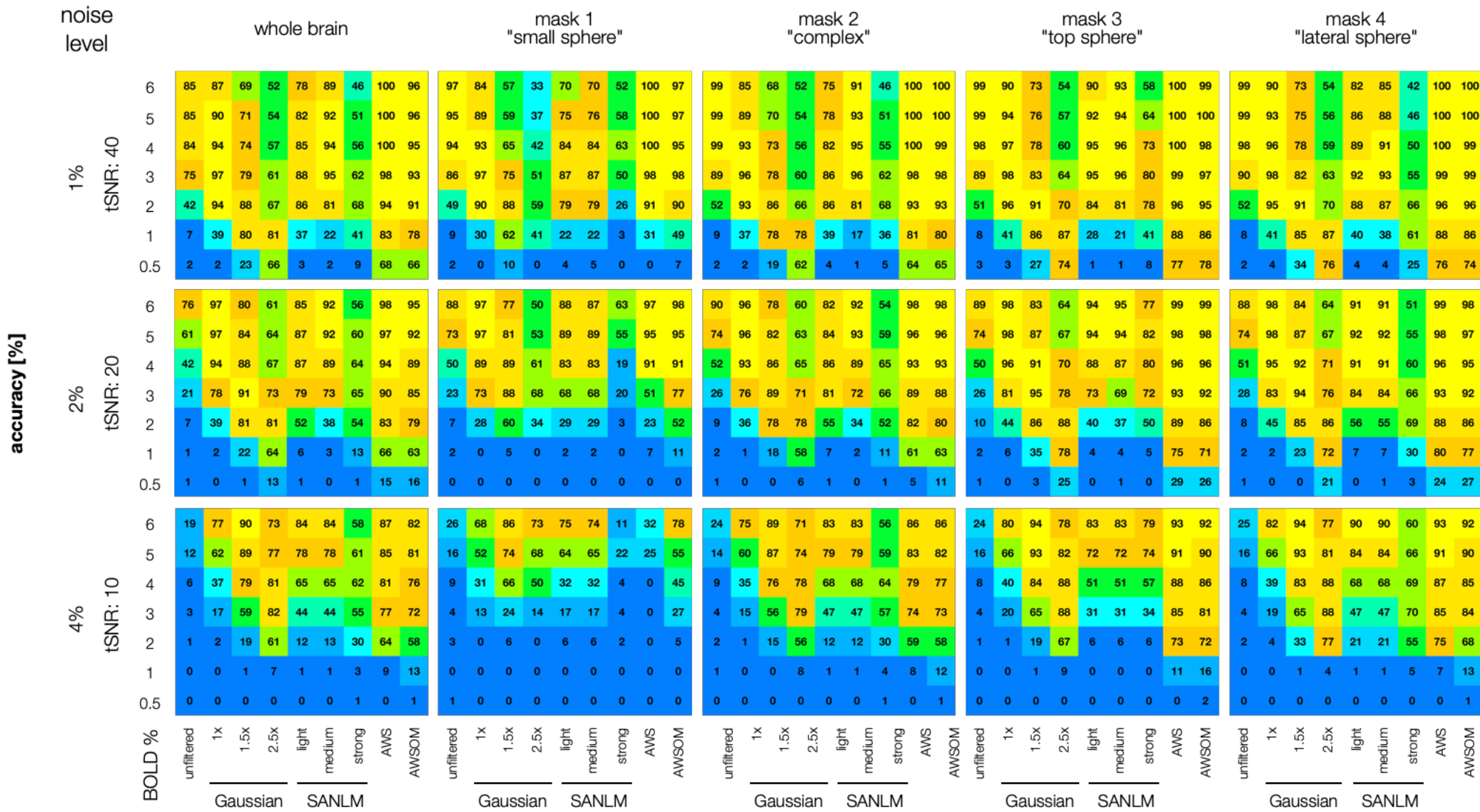

Supplement: Supplementary file 5 — SUPPLEMENTARY FIGURE 5. Accuracy evaluated on simulated fMRI data sets using 7 homogeneous BOLD magnitudes (0.5–6%) and three noise levels (1, 2, 4%) with Rician noise distribution. The average of 10 simulations was calculated for each case. AWS and AWSOM show dominance in each of the conditions, except in the small cluster (mask 1), where Gaussian 1.5x performed best, followed by AWSOM. [file HBM-45-e26813-s002.pdf]

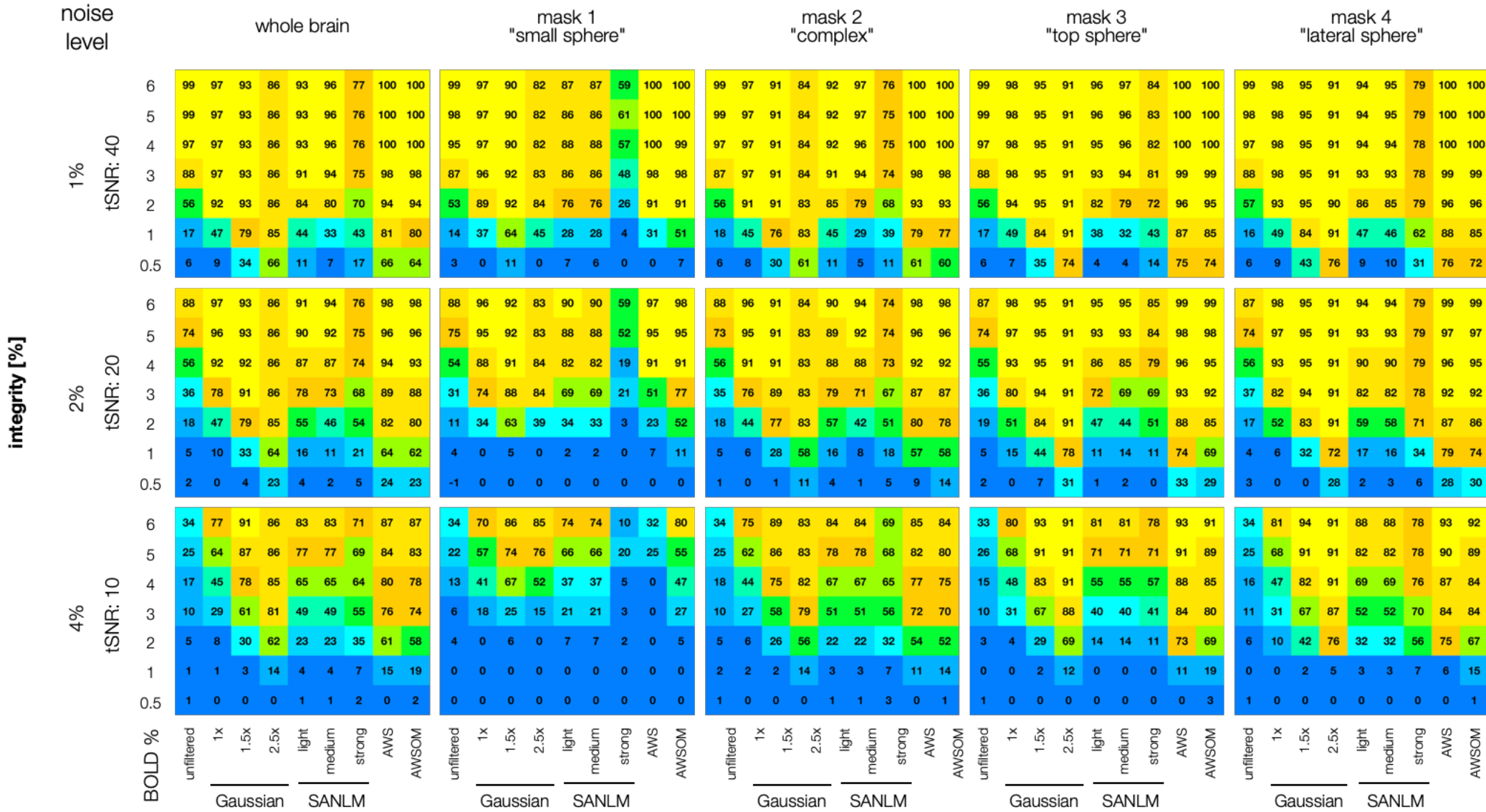

Supplement: Supplementary file 6 — SUPPLEMENTARY FIGURE 6. Signal integrity was measured on 7 homogeneous BOLD magnitudes (0.5–6%) and three noise levels (1, 2, 4%) with Rician noise distribution. BOLD magnitudes of 0.5% were too small to be distinguished from noise, even after filtering, resulting in very low values for all filters. AWSOM was superior in preserving the integrity of the BOLD signal. [file HBM-45-e26813-s009.pdf]
