# Supplementary material for: Precision fMRI and cluster‐failure in the individual brain
Source: Hum Brain Mapp. 2024 Aug 26;45(12):e26813. doi: 10.1002/hbm.26813 (PMC11345700; doi:10.1002/hbm.26813)
Supplement: Supplementary file 7 — SUPPLEMENTARY FIGURE 7. Spatial accuracy, BOLD sensitivity, and signal integrity were evaluated on simulated fMRI data sets using heterogeneous BOLD magnitudes (1.5–6%) and three noise levels (1%, 2%, and 4%) with Rician noise distribution. Each axis corresponds to the analysis performed, which are the separate masks and the whole brain. The plotted lines correspond to the metrics spatial accuracy, BOLD sensitivity and signal integrity. As in the simulations with Gaussian noise distribution, AWSOM was the most balanced for all noise levels. [file HBM-45-e26813-s007.pdf]

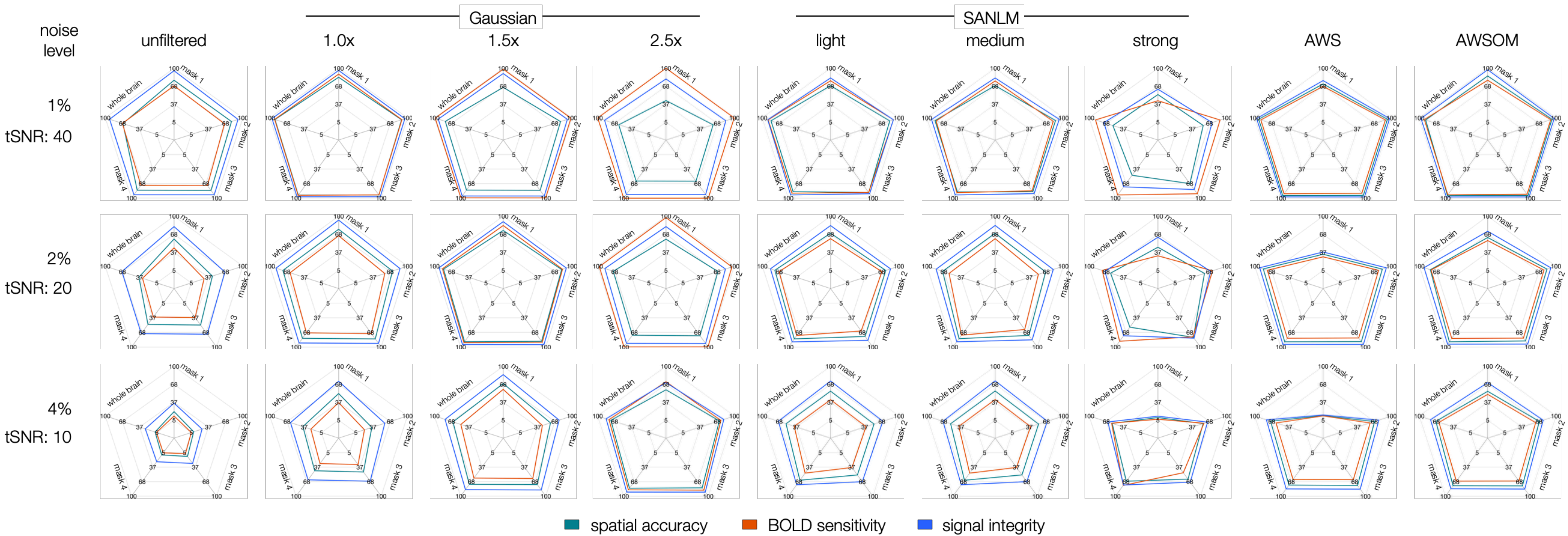

Gaussian
SANLM
noise level
unfiltered
1.0x
1.5x
2.5x
light
medium
strong
AWS
AWSOM
1%
tSNR: 40
2%
tSNR: 20
4%
tSNR: 10
whole brain
mask 1
mask 2
mask 3
mask 4
100
68
37
5
spatial accuracy
BOLD sensitivity
signal integrity
