# Supplementary material for: Precision fMRI and cluster‐failure in the individual brain
Source: Hum Brain Mapp. 2024 Aug 26;45(12):e26813. doi: 10.1002/hbm.26813 (PMC11345700; doi:10.1002/hbm.26813)
Supplement: Supplementary file 8 — SUPPLEMENTARY FIGURE 8. (A) Correlation coefficient heatmaps between Contrast Parameter Estimates (COPE) maps and z maps for homogeneous time series with Gaussian distributed noise. (B) Correlation coefficient between COPE maps and z maps for homogeneous time series with Rician distributed noise. The correlation coefficients were multiplied by a 100 to display the results in percentage. Gaussian filters displayed a nearly perfect correlation in low noise conditions and high BOLD magnitudes. On the other hand, AWS methods depict an extreme low correlation showing that its algorithm handles these parametric maps differently and that they cannot be used interchangeably. [file HBM-45-e26813-s008.pdf]

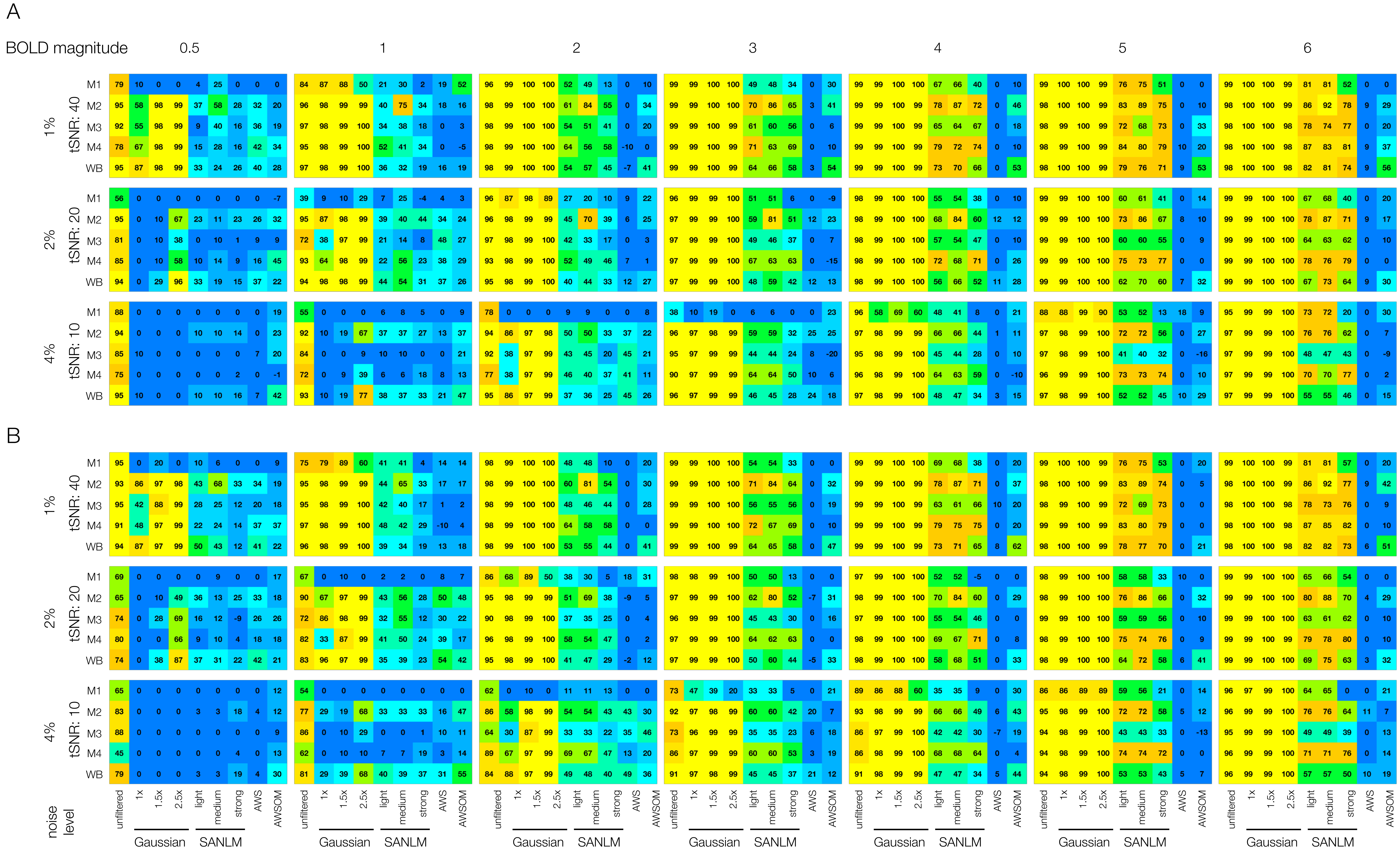
A
B
BOLD magnitude
0.5
1
2
3
4
5
6
1%
tSNR: 40
2%
tSNR: 20
4%
tSNR: 10
M1
M2
M3
M4
WB
noise level
unfiltered
1x
1.5x
2.5x
light
medium
strong
AWS
AWSOM
Gaussian
SANLM
