# Supplementary material for: Precision fMRI and cluster‐failure in the individual brain
Source: Hum Brain Mapp. 2024 Aug 26;45(12):e26813. doi: 10.1002/hbm.26813 (PMC11345700; doi:10.1002/hbm.26813)
Supplement: Supplementary file 9 — SUPPLEMENTARY FIGURE 9. (A) Correlation coefficient heatmaps between COPE maps and z maps for heterogeneous time series with Gaussian distributed noise. (B) Correlation coefficient between COPE maps and z maps for heterogeneous time series with Rician distributed noise. The correlation coefficients were multiplied by a 100 to display the results in percentage. Again, Gaussian filters had a high correlation between maps. The SANLM filters had an intermediate level of correlation, with the strong intensity having the lowest correlation. AWS and AWSOM reached the lowest values, indicating almost zero correlation. [file HBM-45-e26813-s006.pdf]

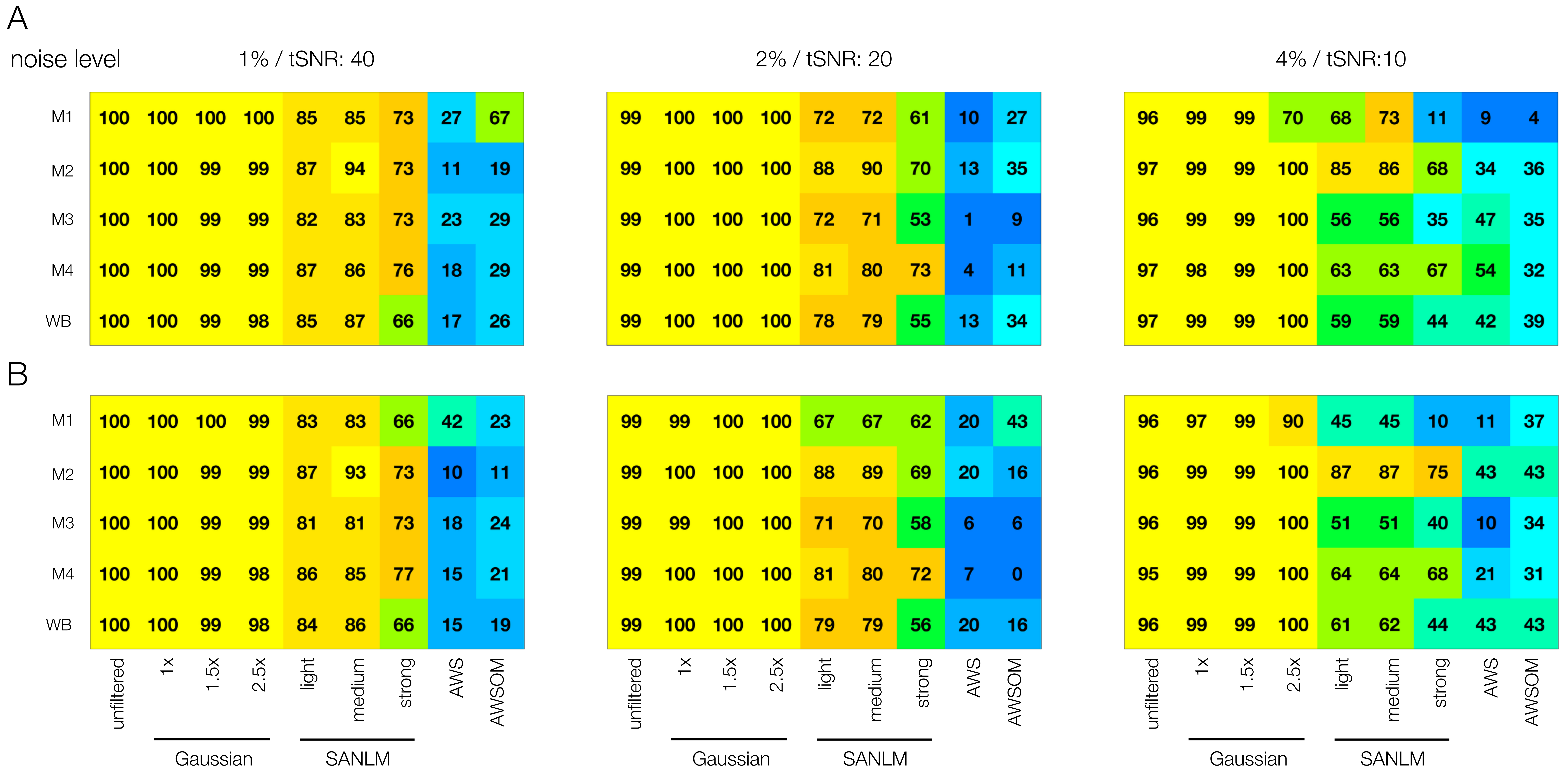
A
noise level
1% / tSNR: 40
2% / tSNR: 20
4% / tSNR:10
M1 100 100 100 100 85 85 73 27 67
M2 100 100 99 99 87 94 73 11 19
M3 100 100 99 99 82 83 73 23 29
M4 100 100 99 99 87 86 76 18 29
WB 100 100 99 98 85 87 66 17 26
99 100 100 100 72 72 61 10 27
99 100 100 100 88 90 70 13 35
99 100 100 100 72 71 53 1 9
99 100 100 100 81 80 73 4 11
99 100 100 100 78 79 55 13 34
96 99 99 70 68 73 11 9 4
97 99 99 100 85 86 68 34 36
96 99 99 100 56 56 35 47 35
97 98 99 100 63 63 67 54 32
97 99 99 100 59 59 44 42 39
B
M1 100 100 100 99 83 83 66 42 23
M2 100 100 99 99 87 93 73 10 11
M3 100 100 99 99 81 81 73 18 24
M4 100 100 99 98 86 85 77 15 21
WB 100 100 99 98 84 86 66 15 19
99 99 100 100 67 67 62 20 43
99 100 100 100 88 89 69 20 16
99 99 100 100 71 70 58 6 6
99 100 100 100 81 80 72 7 0
99 100 100 100 79 79 56 20 16
96 97 99 90 45 45 10 11 37
96 99 99 100 87 87 75 43 43
96 99 99 100 51 51 40 10 34
95 99 99 100 64 64 68 21 31
96 99 99 100 61 62 44 43 43
unfiltered
1x
1.5x
2.5x
light
medium
strong
AWS
AWSOM
Gaussian
SANLM
