# Supplementary material for: Precision fMRI and cluster‐failure in the individual brain
Source: Hum Brain Mapp. 2024 Aug 26;45(12):e26813. doi: 10.1002/hbm.26813 (PMC11345700; doi:10.1002/hbm.26813)
Supplement: Supplementary file 1 — SUPPLEMENTARY FIGURE 1. Sensitivity evaluated on simulated fMRI data sets using 7 homogeneous BOLD magnitudes (0.5–6%) and three noise levels (1, 2, 4%) with Gaussian noise distribution. The average of 10 simulations was calculated for each case. The values in these heat maps correspond to the results shown in the main Figure 4. [file HBM-45-e26813-s005.pdf]

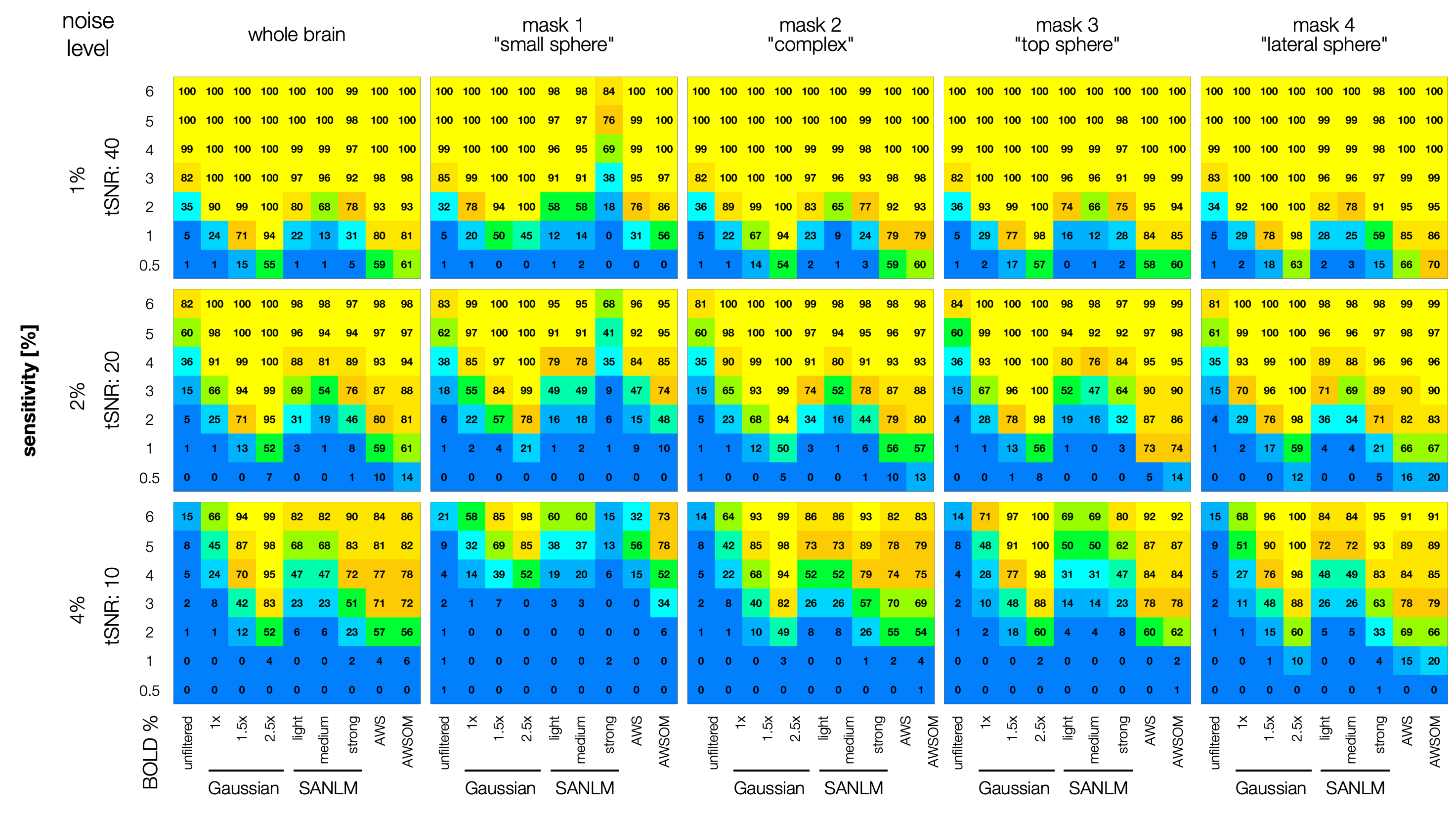
noise level
whole brain
mask 1 "small sphere"
mask 2 "complex"
mask 3 "top sphere"
mask 4 "lateral sphere"
sensitivity [%]
1% tSNR: 40
6 100 100 100 100 100 100 99 100 100 | 100 100 100 100 98 98 84 100 100 | 100 100 100 100 100 100 99 100 100 | 100 100 100 100 100 100 100 100 100 | 100 100 100 100 100 100 98 100 100
5 100 100 100 100 100 100 98 100 100 | 100 100 100 100 97 97 76 99 100 | 100 100 100 100 100 100 99 100 100 | 100 100 100 100 100 100 98 100 100 | 100 100 100 100 99 99 98 100 100
4 99 100 100 100 99 99 97 100 100 | 99 100 100 100 96 95 69 99 100 | 99 100 100 100 99 99 98 100 100 | 99 100 100 100 99 99 97 100 100 | 99 100 100 100 99 99 98 100 100
3 82 100 100 100 97 96 92 98 98 | 85 99 100 100 91 91 38 95 97 | 82 100 100 100 97 96 93 98 98 | 82 100 100 100 96 96 91 99 99 | 83 100 100 100 96 96 97 99 99
2 35 90 99 100 80 68 78 93 93 | 32 78 94 100 58 58 18 76 86 | 36 89 99 100 83 65 77 92 93 | 36 93 99 100 74 66 75 95 94 | 34 92 100 100 82 78 91 95 95
1 5 24 71 94 22 13 31 80 81 | 5 20 50 45 12 14 0 31 56 | 5 22 67 94 23 9 24 79 79 | 5 29 77 98 16 12 28 84 85 | 5 29 78 98 28 25 59 85 86
0.5 1 1 15 55 1 1 5 59 61 | 1 1 0 0 1 2 0 0 0 | 1 1 14 54 2 1 3 59 60 | 1 2 17 57 0 1 2 58 60 | 1 2 18 63 2 3 15 66 70
2% tSNR: 20
6 82 100 100 100 98 98 97 98 98 | 83 99 100 100 95 95 68 96 95 | 81 100 100 100 99 98 98 98 98 | 84 100 100 100 98 98 97 99 99 | 81 100 100 100 98 98 98 99 99
5 60 98 100 100 96 94 94 97 97 | 62 97 100 100 91 91 41 92 95 | 60 98 100 100 97 94 95 96 97 | 60 99 100 100 94 92 92 97 98 | 61 99 100 100 96 96 97 98 97
4 36 91 99 100 88 81 89 93 94 | 38 85 97 100 79 78 35 84 85 | 35 90 99 100 91 80 91 93 93 | 36 93 100 100 80 76 84 95 95 | 35 93 99 100 89 88 96 96 96
3 15 66 94 99 69 54 76 87 88 | 18 55 84 99 49 49 9 47 74 | 15 65 93 99 74 52 78 87 88 | 15 67 96 100 52 47 64 90 90 | 15 70 96 100 71 69 89 90 90
2 5 25 71 95 31 19 46 80 81 | 6 22 57 78 16 18 6 15 48 | 5 23 68 94 34 16 44 79 80 | 4 28 78 98 19 16 32 87 86 | 4 29 76 98 36 34 71 82 83
1 1 1 13 52 3 1 8 59 61 | 1 2 4 21 1 2 1 9 10 | 1 1 12 50 3 1 6 56 57 | 1 1 13 56 1 0 3 73 74 | 1 2 17 59 4 4 21 66 67
0.5 0 0 0 7 0 0 1 10 14 | 0 0 0 0 0 0 0 0 0 | 1 0 0 5 0 0 1 10 13 | 0 0 1 8 0 0 0 5 14 | 0 0 0 12 0 0 5 16 20
4% tSNR: 10
6 15 66 94 99 82 82 90 84 86 | 21 58 85 98 60 60 15 32 73 | 14 64 93 99 86 86 93 82 83 | 14 71 97 100 69 69 80 92 92 | 15 68 96 100 84 84 95 91 91
5 8 45 87 98 68 68 83 81 82 | 9 32 69 85 38 37 13 56 78 | 8 42 85 98 73 73 89 78 79 | 8 48 91 100 50 50 62 87 87 | 9 51 90 100 72 72 93 89 89
4 5 24 70 95 47 47 72 77 78 | 4 14 39 52 19 20 6 15 52 | 5 22 68 94 52 52 79 74 75 | 4 28 77 98 31 31 47 84 84 | 5 27 76 98 48 49 83 84 85
3 2 8 42 83 23 23 51 71 72 | 2 1 7 0 3 3 0 0 34 | 2 8 40 82 26 26 57 70 69 | 2 10 48 88 14 14 23 78 78 | 2 11 48 88 26 26 63 78 79
2 1 1 12 52 6 6 23 57 56 | 1 0 0 0 0 0 0 0 6 | 1 1 10 49 8 8 26 55 54 | 1 2 18 60 4 4 8 60 62 | 1 1 15 60 5 5 33 69 66
1 0 0 0 4 0 0 2 4 6 | 1 0 0 0 0 0 2 0 0 | 0 0 0 3 0 0 1 2 4 | 0 0 0 2 0 0 0 0 2 | 0 0 1 10 0 0 4 15 20
0.5 0 0 0 0 0 0 0 0 0 | 1 0 0 0 0 0 0 0 0 | 0 0 0 0 0 0 0 0 1 | 0 0 0 0 0 0 0 0 1 | 0 0 0 0 0 0 1 0 0
BOLD %
unfiltered 1x 1.5x 2.5x light medium strong AWS AWSOM
Gaussian
SANLM
