# Supplementary material for: Precision fMRI and cluster‐failure in the individual brain
Source: Hum Brain Mapp. 2024 Aug 26;45(12):e26813. doi: 10.1002/hbm.26813 (PMC11345700; doi:10.1002/hbm.26813)
Supplement: Supplementary file 4 — SUPPLEMENTARY FIGURE 4. Sensitivity evaluated on simulated fMRI data sets using 7 homogeneous BOLD magnitudes (0.5–6%) and three noise levels (1, 2, 4%) with Rician noise distribution. The performance of all filters is comparably high at 1 and 2% noise levels. [file HBM-45-e26813-s003.pdf]

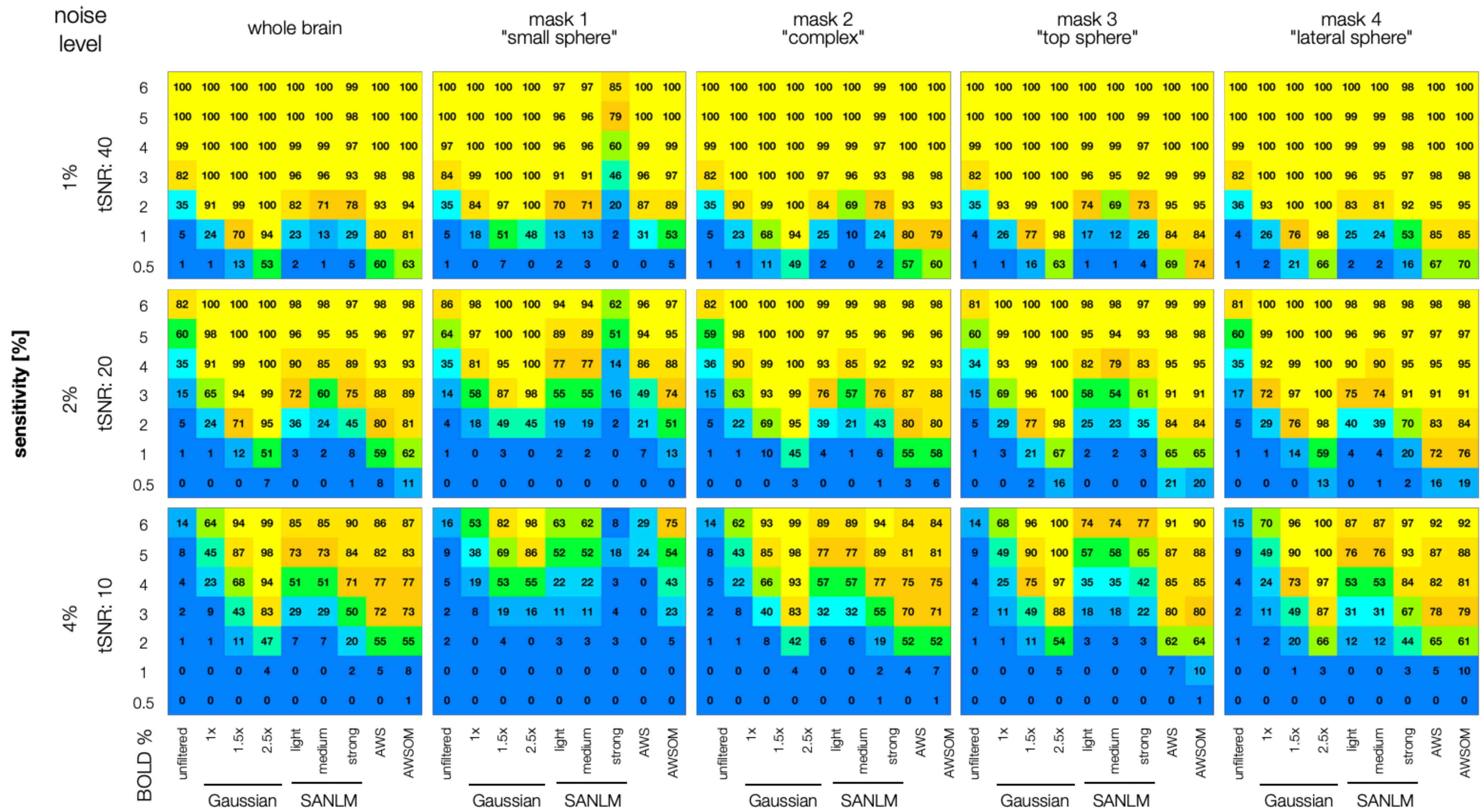
noise level
whole brain
mask 1 "small sphere"
mask 2 "complex"
mask 3 "top sphere"
mask 4 "lateral sphere"
sensitivity [%]
1% tSNR: 40
2% tSNR: 20
4% tSNR: 10
BOLD %
6
5
4
3
2
1
0.5
unfiltered
1x
1.5x
2.5x
light
medium
strong
AWS
AWSOM
Gaussian
SANLM
1% whole brain: 6: 100 100 100 100 100 100 99 100 100
5: 100 100 100 100 100 100 98 100 100
4: 99 100 100 100 99 99 97 100 100
3: 82 100 100 100 96 96 93 98 98
2: 35 91 99 100 82 71 78 93 94
1: 5 24 70 94 23 13 29 80 81
0.5: 1 1 13 53 2 1 5 60 63
1% mask 1: 6: 100 100 100 100 97 97 85 100 100
5: 100 100 100 100 96 96 79 100 100
4: 97 100 100 100 96 96 60 99 99
3: 84 99 100 100 91 91 46 96 97
2: 35 84 97 100 70 71 20 87 89
1: 5 18 51 48 13 13 2 31 53
0.5: 1 0 7 0 2 3 0 0 5
1% mask 2: 6: 100 100 100 100 100 100 99 100 100
5: 100 100 100 100 100 100 99 100 100
4: 99 100 100 100 99 99 97 100 100
3: 82 100 100 100 97 96 93 98 98
2: 35 90 99 100 84 69 78 93 93
1: 5 23 68 94 25 10 24 80 79
0.5: 1 1 11 49 2 0 2 57 60
1% mask 3: 6: 100 100 100 100 100 100 100 100 100
5: 100 100 100 100 100 100 99 100 100
4: 99 100 100 100 99 99 97 100 100
3: 82 100 100 100 96 95 92 99 99
2: 35 93 99 100 74 69 73 95 95
1: 4 26 77 98 17 12 26 84 84
0.5: 1 1 16 63 1 1 4 69 74
1% mask 4: 6: 100 100 100 100 100 100 98 100 100
5: 100 100 100 100 99 99 98 100 100
4: 99 100 100 100 99 99 98 100 100
3: 82 100 100 100 96 95 97 98 98
2: 36 93 100 100 83 81 92 95 95
1: 4 26 76 98 25 24 53 85 85
0.5: 1 2 21 66 2 2 16 67 70
2% whole brain: 6: 82 100 100 100 98 98 97 98 98
5: 60 98 100 100 96 95 95 96 97
4: 35 91 99 100 90 85 89 93 93
3: 15 65 94 99 72 60 75 88 89
2: 5 24 71 95 36 24 45 80 81
1: 1 1 12 51 3 2 8 59 62
0.5: 0 0 0 7 0 0 1 8 11
2% mask 1: 6: 86 98 100 100 94 94 62 96 97
5: 64 97 100 100 89 89 51 94 95
4: 35 81 95 100 77 77 14 86 88
3: 14 58 87 98 55 55 16 49 74
2: 4 18 49 45 19 19 2 21 51
1: 1 0 3 0 2 2 0 7 13
0.5: 0 0 0 0 0 0 0 0 0
2% mask 2: 6: 82 100 100 100 99 99 98 98 98
5: 59 98 100 100 97 95 96 96 96
4: 36 90 99 100 93 85 92 92 93
3: 15 63 93 99 76 57 76 87 88
2: 5 22 69 95 39 21 43 80 80
1: 1 1 10 45 4 1 6 55 58
0.5: 0 0 0 3 0 0 1 3 6
2% mask 3: 6: 81 100 100 100 98 98 97 99 99
5: 60 99 100 100 95 94 93 98 98
4: 34 93 99 100 82 79 83 95 95
3: 15 69 96 100 58 54 61 91 91
2: 5 29 77 98 25 23 35 84 84
1: 1 3 21 67 2 2 3 65 65
0.5: 0 0 2 16 0 0 0 21 20
2% mask 4: 6: 81 100 100 100 98 98 98 98 98
5: 60 99 100 100 96 96 97 97 97
4: 35 92 99 100 90 90 95 95 95
3: 17 72 97 100 75 74 91 91 91
2: 5 29 76 98 40 39 70 83 84
1: 1 1 14 59 4 4 20 72 76
0.5: 0 0 0 13 0 1 2 16 19
4% whole brain: 6: 14 64 94 99 85 85 90 86 87
5: 8 45 87 98 73 73 84 82 83
4: 4 23 68 94 51 51 71 77 77
3: 2 9 43 83 29 29 50 72 73
2: 1 1 11 47 7 7 20 55 55
1: 0 0 0 4 0 0 2 5 8
0.5: 0 0 0 0 0 0 0 0 1
4% mask 1: 6: 16 53 82 98 63 62 8 29 75
5: 9 38 69 86 52 52 18 24 54
4: 5 19 53 55 22 22 3 0 43
3: 2 8 19 16 11 11 4 0 23
2: 2 0 4 0 3 3 3 0 5
1: 0 0 0 0 0 0 0 0 0
0.5: 0 0 0 0 0 0 0 0 0
4% mask 2: 6: 14 62 93 99 89 89 94 84 84
5: 8 43 85 98 77 77 89 81 81
4: 5 22 66 93 57 57 77 75 75
3: 2 8 40 83 32 32 55 70 71
2: 1 1 8 42 6 6 19 52 52
1: 0 0 0 4 0 0 2 4 7
0.5: 0 0 0 0 0 0 1 0 1
4% mask 3: 6: 14 68 96 100 74 74 77 91 90
5: 9 49 90 100 57 58 65 87 88
4: 4 25 75 97 35 35 42 85 85
3: 2 11 49 88 18 18 22 80 80
2: 1 1 11 54 3 3 3 62 64
1: 0 0 0 5 0 0 0 7 10
0.5: 0 0 0 0 0 0 0 0 1
4% mask 4: 6: 15 70 96 100 87 87 97 92 92
5: 9 49 90 100 76 76 93 87 88
4: 4 24 73 97 53 53 84 82 81
3: 2 11 49 87 31 31 67 78 79
2: 1 2 20 66 12 12 44 65 61
1: 0 0 1 3 0 0 3 5 10
0.5: 0 0 0 0 0 0 0 0 0
